# Empirical Calibration of RGB Vegetation Indices for Non-Destructive Shoot Segmentation and Area Estimation in Blackberry Micropropagation

**DOI:** 10.3390/plants15213460

**Published:** 2026-11-06

**Authors:** Raffaella Brigante, Laura Marconi, Luca Regni

**Affiliations:** 1 Department of Engineering, University of Perugia, 06125 Perugia, Italy; raffaella.brigante@unipg.it (R.B.); laura.marconi@unipg.it (L.M.); 2 Department of Agricultural, Food and Environmental Sciences, University of Perugia, 06121 Perugia, Italy

**Keywords:** RGB vegetation indices, image segmentation, micropropagation, *Rubus* spp., Structure-from-Motion photogrammetry, ExG, GLI

## Abstract

Accurate, non-destructive quantification of shoot development in plant micropropagation is essential for optimising culture protocols and improving throughput. This study evaluated the performance of four RGB vegetation indices, Excess Green (ExG), Green Leaf Index (GLI), Modified Green Red Vegetation Index (MGRVI), and Visible Atmospherically Resistant Index (VARI), for the automated segmentation of *Rubus* spp. shoots in close-range orthophotos acquired with a smartphone camera. Twelve vessels, each containing fifteen blackberry shoots, grown at four sucrose concentrations (0, 7.5, 15 and 30 g L^−1^) were analysed at both pixel and ROI (Region Of Interest) levels. Classification thresholds were determined by maximising the Youden index on the ROC curve, both as a single global threshold and as concentration-specific thresholds. GLI and ExG substantially outperformed MGRVI and VARI in all discriminability metrics (AUC-ROC = 0.991 and 0.988, respectively), with stable classification performance across all sucrose concentrations. Projected shoot cluster area estimates derived from the two best-performing indices were benchmarked against a geometrically explicit reference obtained from a colour-filtered 3D point cloud reconstructed by Structure-from-Motion (SfM) photogrammetry from the same image set. Strong agreement was observed between RGB-based and point-cloud-derived area estimates ($R^{2}=0.990$ for GLI), with Intersection over Union (IoU) ≥0.84 for all vessels. Concentration-specific thresholds provided a modest improvement in in-sample regression fit over the single global threshold without substantially affecting geometric agreement. A separate external dataset of six vessels, with independently annotated masks, was subsequently used to assess threshold transferability (mean IoU 0.908 for GLI and 0.861 for ExG). The results indicate that, under the tested conditions, GLI and ExG applied to smartphone-acquired orthophotos provide a relatively low-cost, non-destructive approach for projected shoot cluster area extraction in *Rubus* spp. in vitro cultures. The study is primarily methodological: the relationship between the image-derived measurements and independent biological descriptors of shoot development or propagation performance was not assessed.

## 1. Introduction

Belonging to the Rosaceae family, the blackberry (*Rubus* spp.) represents a fruit crop of considerable economic importance on a global scale. Its appeal in international markets stems largely from a nutritional profile rich in bioactive molecules which confer notable antioxidant properties and help counteract oxidative stress and inflammatory processes [1,2].

Traditional propagation relies heavily on layering and root cuttings, approaches that demand large cultivation areas, substantial labor input, and ongoing weed control [3].

Against this backdrop, in vitro culture has become established as the leading alternative for blackberry propagation [3]. Its principal advantage lies in the ability to generate, rapidly and on a large scale, plant material that is both genetically homogeneous [4,5] and pathogen-free, a feature valuable both for commercial nurseries and for germplasm preservation efforts [1,6,7,8].

In vitro culture of Rubus was first reported roughly fifty years ago [9], with blackberry-specific protocols established shortly after [10,11]. Decades of subsequent optimisation [12] have made it the method of choice for commercial-scale blackberry propagation [3].

Despite this widespread optimisation of micropropagation protocols, culture performance remains highly sensitive to medium composition, and the carbon source in particular, most commonly sucrose, plays a dual role as both the primary energy substrate under limited photosynthetic capacity and as an osmotic regulator influencing morphogenesis within the culture vessel [13,14,15]. Optimising such parameters requires the ability to monitor shoot growth accurately and repeatedly across treatments. Yet, as recently highlighted by Bethge et al. [16], the majority of evaluation methods used to describe the performance of in vitro cultures remain based on destructive approaches, limiting data collection to unique endpoint measurements.

Such limitations have motivated, across plant sciences more broadly, a shift toward image-based, non-destructive phenotyping methods capable of extracting quantitative morphological traits with higher throughput and repeatability. As part of the broader shift toward image-based phenotyping, spectral vegetation indices computed directly from digital imagery have become a central tool for translating pixel-level colour or reflectance information into quantitative descriptors of vegetation cover and physiological status.

Among these indices, the Normalized Difference Vegetation Index (NDVI) remains the most widely used metric for characterising vegetation cover, vigour, and canopy dynamics, owing to its strong physiological grounding in chlorophyll absorption and its long-standing validation across ecosystems and crops [17]. However, NDVI computation requires reflectance measurements in the near-infrared region, which are only obtainable with multispectral or hyperspectral sensors, instrumentation considerably more expensive and less accessible than conventional RGB cameras. Similarly, chlorophyll indices incorporating red-edge reflectance (e.g., the Chlorophyll Index Red Edge - CI_Red-edge_ and the MERIS Terrestrial Chlorophyll Index - MTCI) [18,19] have been shown to provide highly accurate and even crop-type-insensitive estimates of green leaf area index [20], but likewise depend on spectroradiometers or multispectral/hyperspectral imaging systems. Colour-based vegetation indices derived from consumer-grade RGB imagery therefore remain the most practical low-cost alternative for vegetation segmentation and canopy monitoring, relying exclusively on the visible spectral bands captured by standard digital cameras, including smartphones and UAV-mounted consumer cameras, across a wide range of platforms and scales [21,22,23].

Several RGB-based indices have been proposed, differing in the specific combination and normalisation of the red, green, and blue channels used to enhance the spectral contrast between vegetation and background, including Excess Green (ExG), Green Leaf Index (GLI), Modified Green Red Vegetation Index (MGRVI), and Visible Atmospherically Resistant Index (VARI) [23,24,25]. However, comparative studies converge on the finding that no single RGB index performs consistently best across acquisition conditions, and that relative index rankings obtained in outdoor, variable-illumination imagery are not necessarily transferable to other acquisition contexts [22,26,27]. Beyond field- and canopy-scale applications, RGB-derived greenness metrics have also been used for finer-scale, controlled-background plant phenotyping [28,29], an application context considerably closer, in terms of imaging scale and controlled background, to the segmentation task addressed in the present study.

Taken together, these studies indicate that while RGB indices have been extensively benchmarked in open-field and UAV-based settings, their application to the segmentation of shoot tissue in the highly controlled, small-scale, close-range imaging conditions typical of in vitro culture vessels has, to our knowledge, rarely been investigated. Such 2D methods, moreover, cannot capture the full spatial organisation of explants and are prone to underestimating growth in vessels where shoots partially overlap or occlude one another when viewed from above. These constraints highlight the need for 3D acquisition methods that are both geometrically reliable and readily deployable within a standard micropropagation laboratory [30,31,32].

Structure-from-Motion (SfM) photogrammetry has emerged as a powerful, low-cost tool for reconstructing three-dimensional plant structure from overlapping 2D images, without the need for specialised or costly 3D sensors [33,34], yielding a metrically accurate three-dimensional point cloud comparable to that obtained via laser scanning. Such 3D reconstruction techniques have been applied across a wide range of scales: at the orchard and field level, point clouds obtained through laser scanning or photogrammetry have been increasingly used to characterise canopy architecture in woody and shrub crops, including species closely related to *Rubus* [35,36]. At the in vitro scale, by contrast, early image-based approaches to growth monitoring relied exclusively on 2D imaging and colour-based physiological indices, without any geometric or volumetric component [37,38,39].

Smartphone-integrated, RTK-enabled positioning systems (e.g., the viDoc RTK Rover, designed to interface with LiDAR-equipped smartphones) have recently lowered the cost and complexity of acquiring accurate, georeferenced 3D reconstructions at close range, with sub-centimetre geometric accuracy validated both in non-agricultural applications [40,41] and directly against Terrestrial Laser Scanning in an arboreal context [42,43].

Direct application to micropropagation has been demonstrated for the first time by Regni et al. [30], who combined an iPhone with the viDoc RTK Rover to reconstruct dense point clouds of blackberry and blueberry explants, relying on a planar (2D) analysis obtained through horizontal projection to extract shoot-covered area and shoot cluster density. Regni et al. [31] subsequently extended the approach to a fully volumetric (3D) shoot-level analysis in kiwifruit explants, deriving height, surface area, volume, and shape indices directly from the point cloud geometry. More recently, Brigante et al. [32] further refined this volumetric framework in blackberry by introducing voxel-based structural descriptors and comparing three complementary volumetric estimators (DSM-based, voxel-based, and convex hull). Although these three studies share the same acquisition hardware (smartphone coupled with the viDoc RTK Rover), in all of them the point cloud itself constitutes the primary source of morphometric measurement. The present study follows a different path: rather than deriving shoot metrics directly from the point cloud, it evaluates a purely 2D, RGB vegetation-index-based segmentation approach computed from ordinary RGB orthophotos, and uses the 3D point cloud exclusively as a geometric reference against which this simpler method is benchmarked. Specifically, this study reuses the same image acquisitions and experimental set-up (cultivar, sucrose treatments, vessel layout, and acquisition protocol) as Brigante et al. [32], which instead derived volumetric shoot descriptors (height, volume, shape indices) directly from the point cloud; the RGB vegetation-index thresholds and classification framework used here were calibrated entirely independently of that analysis.

Taken together, RGB-based vegetation indices, SfM-derived point clouds, and automated multi-sensor phenotyping platforms [16] have each been independently developed and validated for non-destructive plant monitoring, yet no single study has integrated them within the specific context of micropropagation. Their relative performance under close-range, controlled-background, in vitro conditions, and their mutual geometric benchmarking, remains, to our knowledge, largely unexplored prior to Regni et al. [30,31], Brigante et al. [32], on which the present study directly builds.

Based on these results, the objective of the present study was to develop a reliable, empirically calibrated RGB-based method for automated shoot segmentation and area quantification in *Rubus* spp. micropropagation, using ordinary RGB images alone, without recourse to multispectral or hyperspectral acquisition. To this end, four RGB vegetation indices (ExG, GLI, MGRVI, VARI) computed from smartphone-acquired close-range orthophotos, across twelve in vitro culture vessels grown under four sucrose concentrations (0, 7.5, 15 and 30 g L^−1^), were compared to identify the most reliable one for separating shoot tissue from background at both the pixel and whole-vessel levels. A classification threshold was then empirically calibrated and tested for generalisability across sucrose treatments; the resulting area estimates were evaluated against a geometrically explicit reference obtained through SfM-based 3D point cloud reconstruction and colour-filtered planar projection.

The study is therefore primarily methodological in scope: the micropropagation experiment, with its four sucrose concentrations, provides a controlled test set for calibrating and benchmarking RGB-based segmentation, whereas shoot development, propagation performance and culture optimisation were not assessed, and the sucrose-related patterns are reported as exploratory. Overall, this work aims to demonstrate that RGB-based segmentation of SfM-derived orthophotos, without any dedicated multispectral or 3D-sensing hardware, can provide a reliable, low-cost basis for automated shoot segmentation and projected shoot cluster area extraction, with the resulting classification framework potentially applicable in principle to a single, standardised top-view image, a possibility discussed, but not experimentally validated here, in Section 3. The methodological contribution of this study lies not in the acquisition hardware itself, but in the empirically calibrated, ROC/Youden-based segmentation framework, its geometric benchmarking and the external validation, establishing a transferable, quantitatively evaluated protocol rather than a single-crop application of existing tools.

## 2. Results

### 2.1. Descriptive Statistics, Class Separability, and Classification Performance

**Figure 1:**
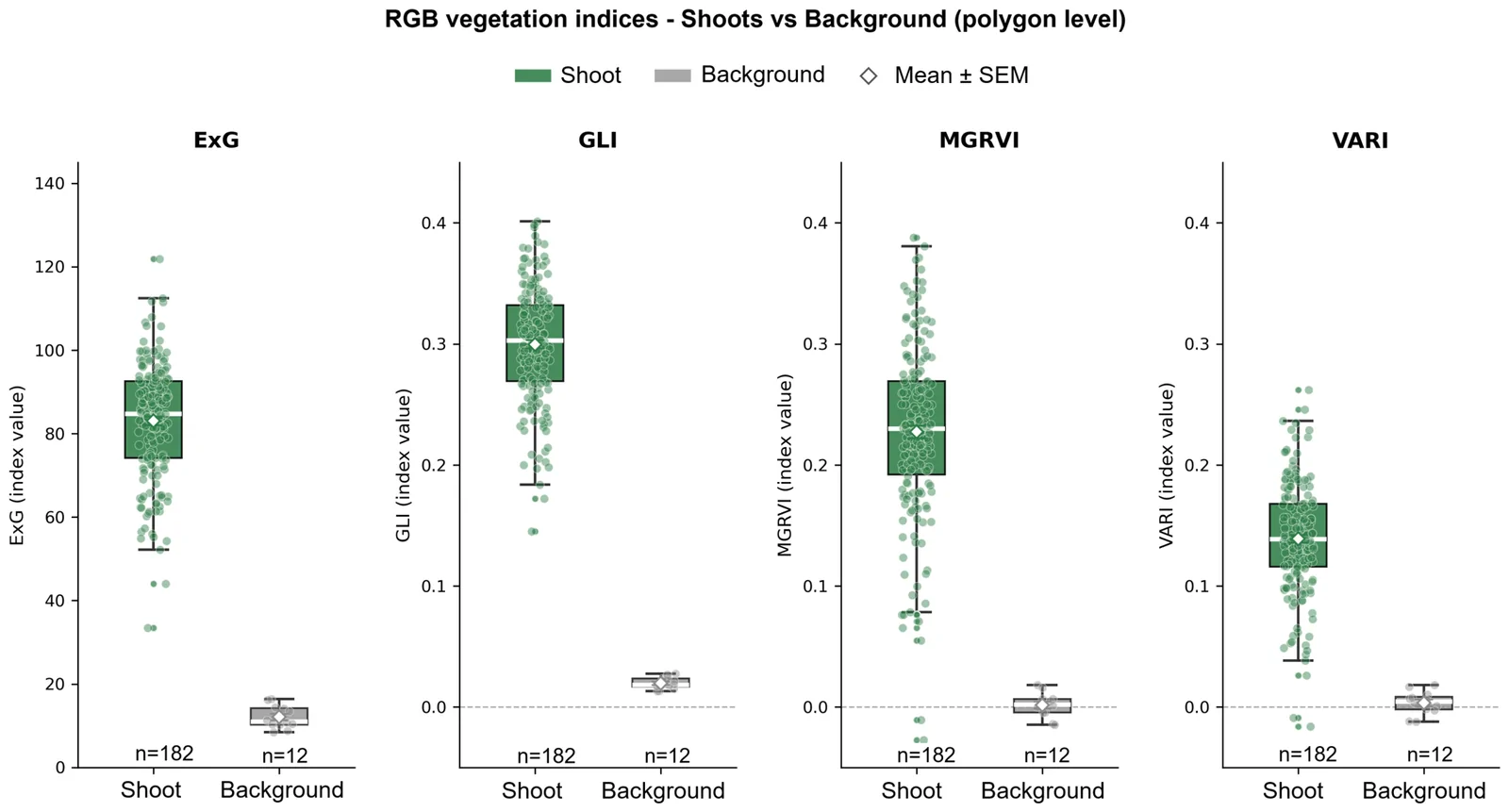
Box-plots of mean polygon values for the four RGB vegetation indices (ExG, GLI, MGRVI, VARI) by class (Shoot cluster: green; Background: grey) at the ROI level. Boxes show the interquartile range (IQR); horizontal white lines indicate medians; whiskers extend to 1.5×IQR.

At the ROI level, descriptive statistics of the four RGB vegetation indices showed a clear separation between the SC and BG classes (Table S1 in Supplementary Materials). ExG showed mean values of 83.04 ± 13.92 for SC polygons and 12.07 ± 2.75 for BG polygons; GLI averaged 0.300 ± 0.048 for SC and 0.020 ± 0.005 for BG. Box-plots of ROI-level index values (Figure 1) confirmed this separation for all four indices, with ExG and GLI showing the widest relative distance between class medians combined with the most compact BG distributions, whereas MGRVI and VARI showed broader BG variability and greater overlap with the lower tail of the SC distribution.

**Figure 2:**
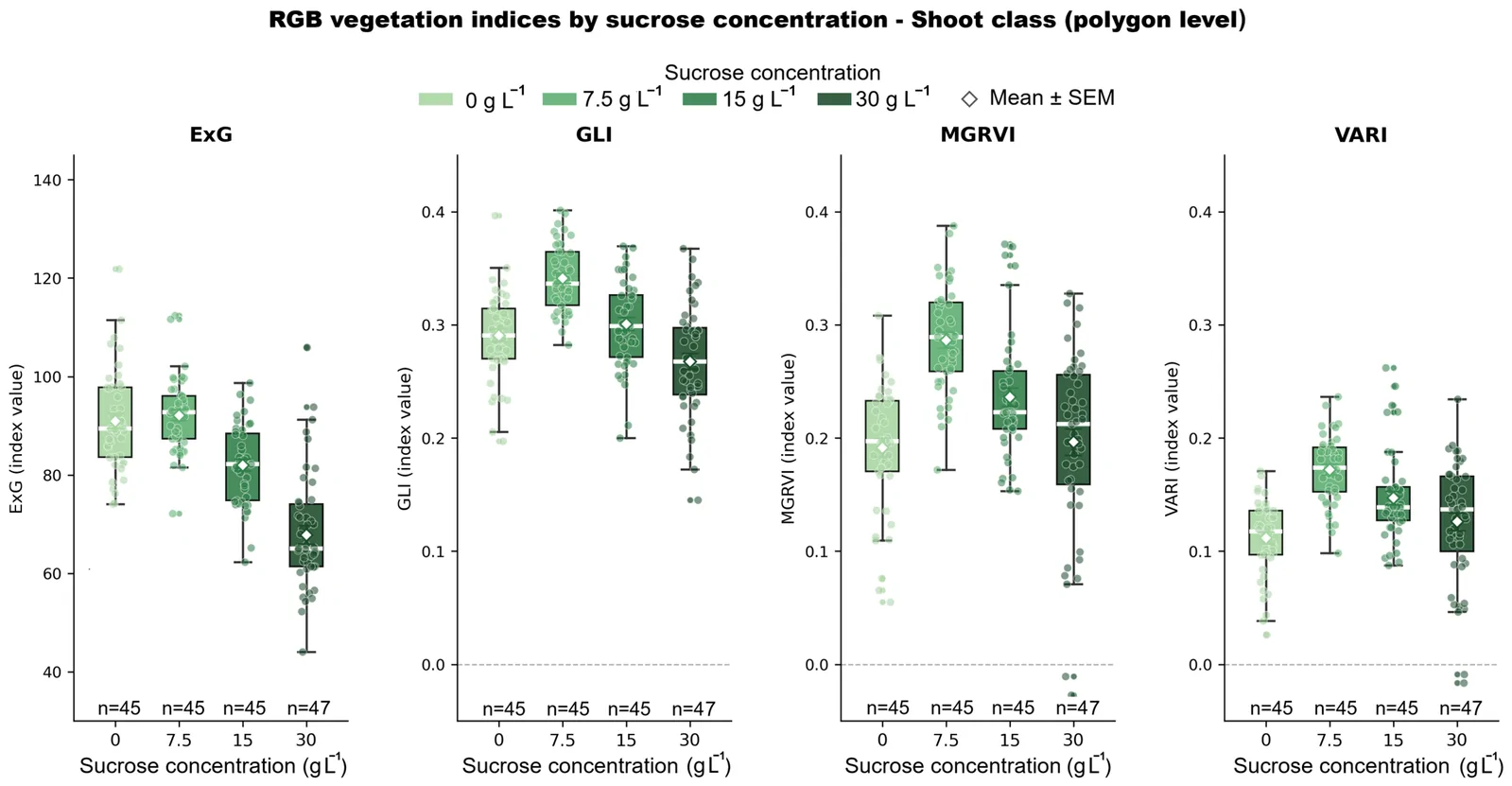
Box-plots of mean polygon index values (ExG, GLI, MGRVI, VARI) for the Shoot cluster class across the four sucrose concentrations (0, 7.5, 15, 30 g L^−1^) at the ROI level. Colour gradient from light green (0 g L^−1^) to dark green (30 g L^−1^). Boxes show the interquartile range (IQR); horizontal white lines indicate medians; whiskers extend to 1.5 × IQR.

Box-plots of mean polygon index values for the SC class revealed a non-monotonic, hump-shaped trend across sucrose concentrations rather than a simple decrease with increasing sucrose (Figure 2): values peaked at 7.5 g L^−1^ rather than at 0 g L^−1^, and then declined progressively at 15 and 30 g L^−1^, the latter treatment consistently showing the lowest values across all four indices. This pattern is examined statistically in Section 2.3.

At the pixel level (*n* = 116,498 sampled points; 92,574 SC, 23,924 BG), descriptive statistics confirmed the separation observed at the ROI level, with mean values consistent between the two spatial scales for the normalised indices, indicating good representativeness of the ROI-level sampling (Figure S1 in Supplementary Materials). As expected, pixel-level standard deviations were markedly higher than ROI level ones (e.g., ExG Shoot: 28.12 vs. 13.92) due to intra-polygon spectral heterogeneity being averaged out at the polygon scale.

**Figure 3:**
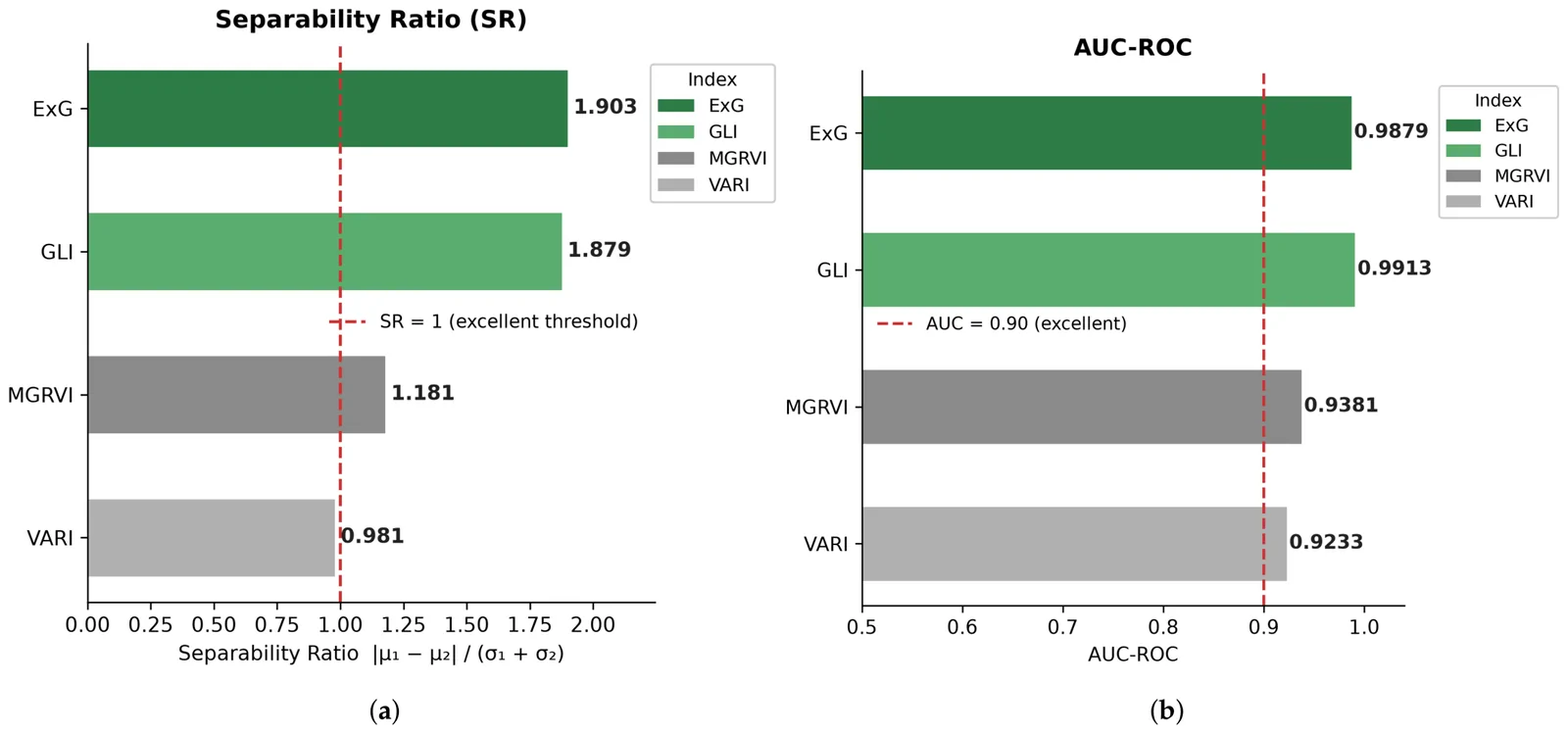
Separability metrics at the pixel level for the four RGB vegetation indices (ExG, GLI, MGRVI, VARI): (**a**) Separability Ratio (SR); (**b**) AUC-ROC. Red dashed lines indicate reference thresholds (SR = 1.0; AUC = 0.90). ExG and GLI (green bars) outperformed MGRVI and VARI (grey bars) on both metrics.

Separability analysis at the pixel level (see Figure 3 and Table S2 in Supplementary Material) showed that ExG and GLI outperformed MGRVI and VARI in both complementary separability metrics considered (SR and AUC−ROC). ExG achieved the highest Separability Ratio (SR=1.903), followed by GLI (SR=1.879), MGRVI (SR=1.181), and VARI (SR=0.981); GLI showed the highest AUC-ROC (0.9913), followed closely by ExG (0.9879), whereas MGRVI (0.9381) and VARI (0.9233) showed comparatively lower discriminative power.

**Figure 4:**
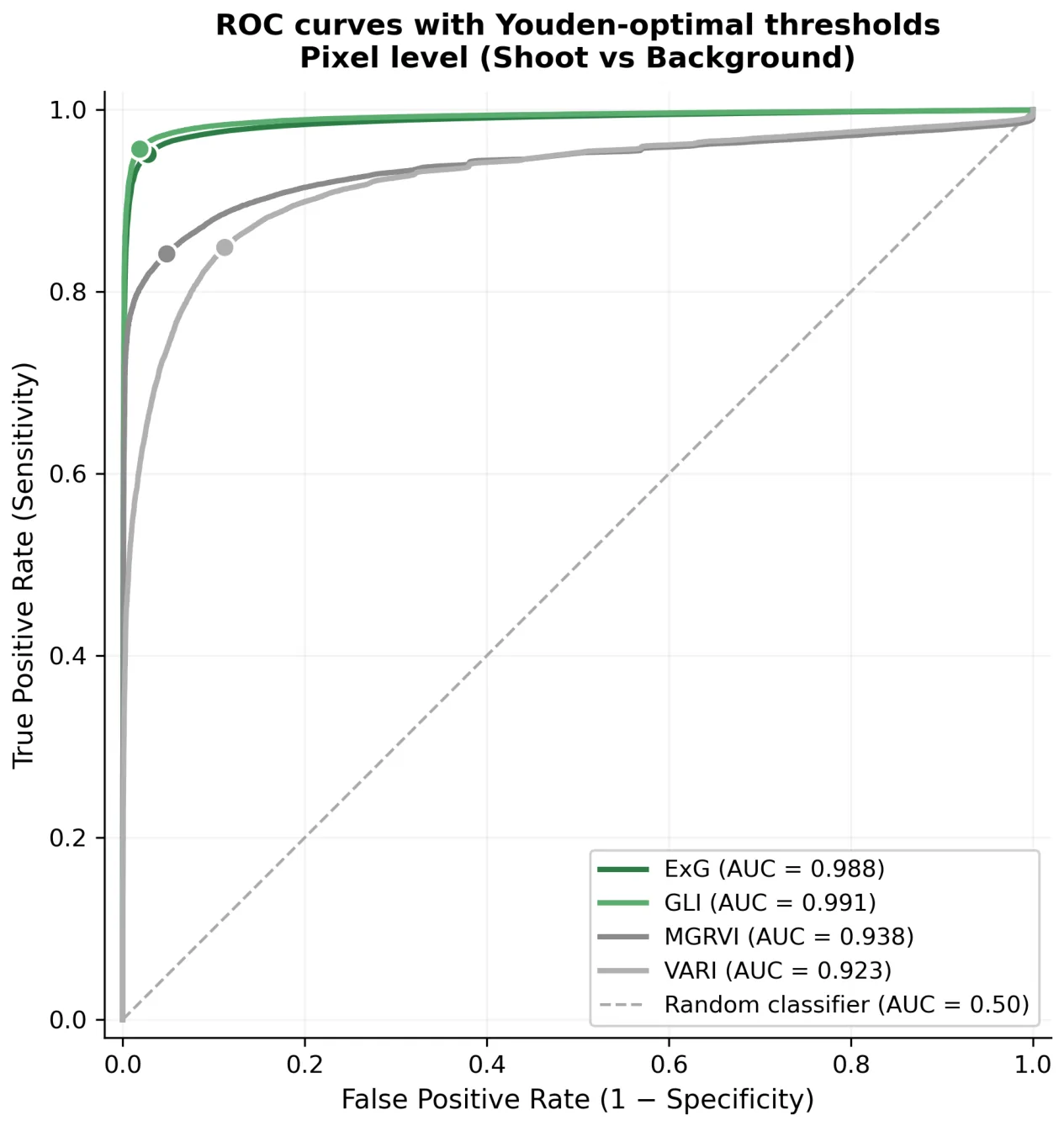
ROC curves for the four RGB vegetation indices (ExG, GLI, MGRVI, VARI) at the pixel level. The dashed diagonal line represents a random classifier (AUC = 0.50). Coloured dots indicate the optimal classification threshold for each index, determined by maximising the Youden index (*J* = Sensitivity + Specificity − 1).

ROC curve analysis (Figure 4) showed that GLI and ExG were the two best-performing indices, with GLI showing a marginally higher AUC (GLI = 0.9913; ExG = 0.9879), while MGRVI (AUC = 0.9381) and VARI (AUC = 0.9233) showed comparatively lower discriminative power. The Youden index identified global optimal classification thresholds of 0.0658 for GLI (sensitivity = 0.957, specificity = 0.981) and 33.0 for ExG (sensitivity = 0.952, specificity = 0.973) (see Table S2 in Supplementary Material).

ROC curve analysis (Figure 4) showed that GLI and ExG were the two best-performing indices, with GLI showing a marginally higher AUC (GLI = 0.9913; ExG = 0.9879), while MGRVI (AUC = 0.9381) and VARI (AUC = 0.9233) showed comparatively lower discriminative power. The Youden index identified global optimal classification thresholds of 0.0658 for GLI (sensitivity = 0.957, specificity = 0.981) and 33.0 for ExG (sensitivity = 0.952, specificity = 0.973) (see Table S2 in Supplementary Material).

**Figure 5:**
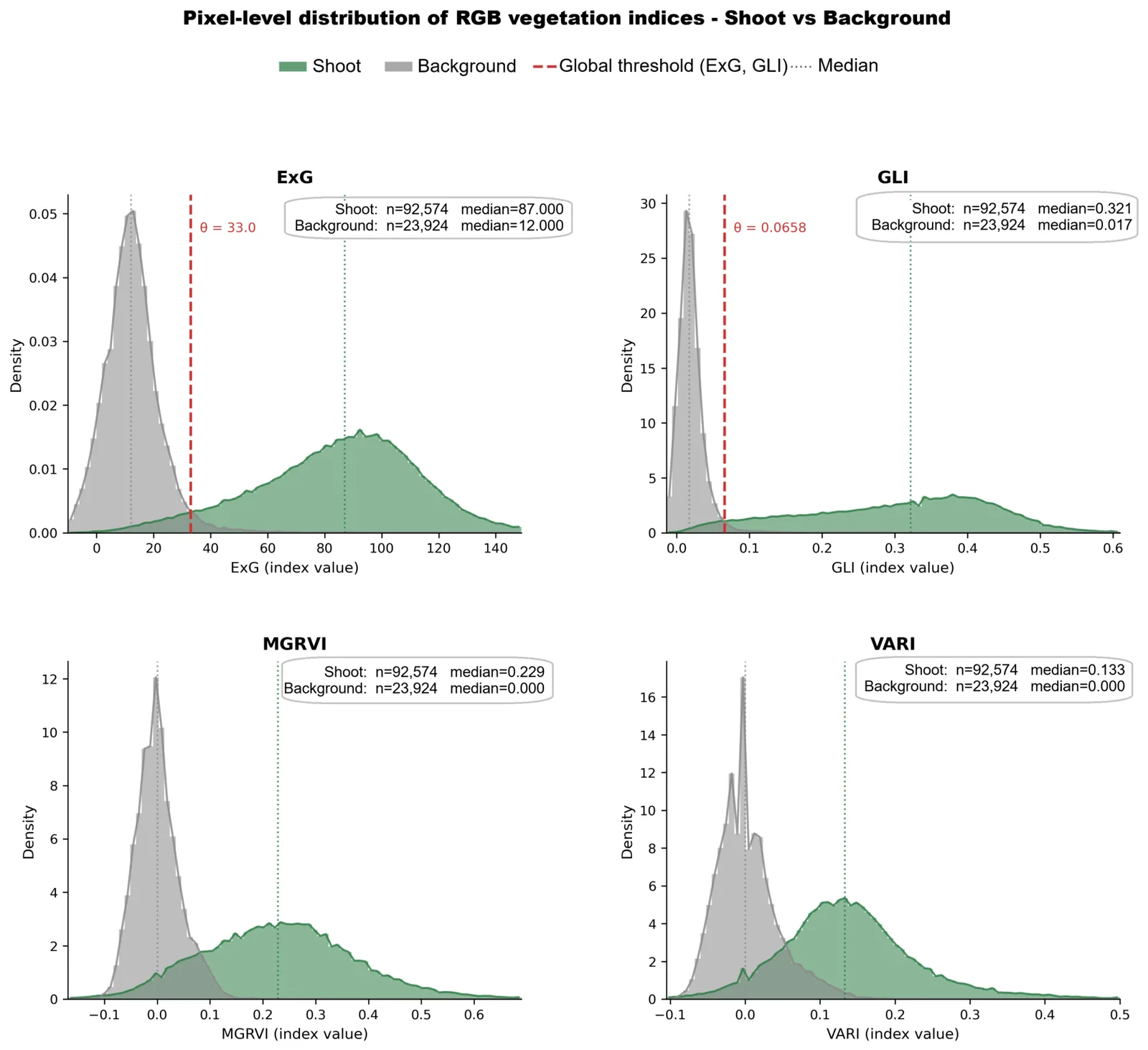
Normalised frequency distributions (density histograms) of pixel-level values for the four RGB vegetation indices (ExG, GLI, MGRVI, VARI) for the Shoot cluster (green, n = 92,574) and Background (grey, n = 23,924) classes. Red dashed vertical lines indicate the global optimal classification thresholds for ExG (θ=33.0) and GLI (θ=0.0658) determined by the Youden index. Dotted vertical lines indicate class medians.

Pixel-level frequency distributions confirmed the separability of SC and BG classes for all four indices (Figure 5), with ExG and GLI showing the least overlap between class distributions. The global optimal thresholds (ExG: θ=33.0; GLI: θ=0.0658), determined by maximising the Youden index on the ROC curve, fell in the region of minimum overlap between the two distributions, indicating effective separation of SC tissue from BG at the pixel scale.

Applying the global Youden-optimal thresholds, GLI achieved the highest classification accuracy (Accuracy = 0.9619, F1 = 0.9756), with sensitivity = 0.9569 and specificity = 0.9812; ExG performed similarly (Accuracy = 0.9558, F1 = 0.9716) (see Table S2 and Figure S2 in Supplementary Material). MGRVI and VARI showed markedly lower accuracy (0.8647 and 0.8569, respectively), driven primarily by reduced sensitivity (0.842 and 0.849), while precision remained high for all indices (>0.96), indicating that classification errors for MGRVI and VARI were dominated by false negatives (i.e., SC pixels misclassified as BG) rather than false positives. These metrics describe classification performance on the calibration dataset.

### 2.2. Stability of Classification Performance Across Sucrose Treatments

To assess whether the discriminative performance of the two best-performing indices depended on sucrose concentration, AUC-ROC was computed separately for ExG and GLI within each of the four treatments (Table 1). AUC-ROC remained consistently high across all sucrose treatments for both indices, with AUC-ROC values ranging from 0.978 to 0.994 for ExG and from 0.987 to 0.995 for GLI, compared to global AUC values of 0.9879 (ExG) and 0.9913 (GLI). A slight reduction was observed at 30 g/L, particularly for ExG (AUC = 0.978), consistent with the greater variability in shoot appearance observed at this concentration in other analyses (see Section 2.3). Overall, this consistency indicates that the classification performance of ExG and GLI is not substantially affected by sucrose concentration, supporting their use for automated shoot segmentation across the tested range of sucrose concentrations without requiring a separate index selection for each treatment. Applying the fixed global thresholds to each treatment subset (Table S6 in Supplementary Material) confirmed robust classification performance across all sucrose concentrations, with accuracy remaining above 0.91 in every case; the largest, though still moderate, reduction occurred at 30 g L^−1^ (ExG accuracy = 0.915), consistent with the AUC-ROC pattern reported above and with the greater variability in shoot appearance observed at this concentration (Section 2.3). At the vessel level (Table S7), specificity was at least 0.942 for GLI and 0.914 for ExG in every vessel (minimum in TF-7.5A). Variation among the three vessels of each treatment was smallest at 15 g L^−1^ (sensitivity ranges ≤0.015) and was driven by single vessels elsewhere: sensitivity ranged from 0.935 to 0.987 (GLI) and from 0.945 to 0.989 (ExG) at 0 g L^−1^, owing to TF-0A, and from 0.913 to 0.946 (GLI) and from 0.865 to 0.916 (ExG) at 30 g L^−1^; specificity ranged from 0.942 to 0.984 (GLI) and from 0.914 to 0.970 (ExG) at 7.5 g L^−1^. Whole-mask IoU ranged from 0.889 to 0.928 for GLI and from 0.848 to 0.921 for ExG across all twelve vessels, with the lowest values at 30 g L^−1^ for ExG.

Leave-one-vessel-out cross-validation confirmed the stability of the calibrated thresholds: across the 12 folds, the Youden-optimal threshold ranged from 32.0 to 33.0 for ExG and from 0.0605 to 0.0658 for GLI, closely matching the globally calibrated values (33.0 and 0.0658, respectively; Table S9 in Supplementary Material). Out-of-fold classification performance (mean ± SD across the 12 held-out vessels) was characterized by: accuracy = 0.956±0.028, sensitivity = 0.951±0.038, specificity = 0.972±0.026 for ExG, and accuracy = 0.962±0.017, sensitivity = 0.958±0.023, specificity = 0.977±0.022 for GLI, essentially indistinguishable from the in-sample performance reported above, indicating that the empirically calibrated thresholds are not overfitted to the specific vessels used for calibration.

### 2.3. Effect of Sucrose Concentration on Index Values

At the vessel level (n=3 per treatment), the Kruskal-Wallis test confirmed statistically significant differences among sucrose concentrations for all four RGB vegetation indices (SC class; ExG: H=9.36, df=3, p=0.025; GLI: H=9.67, p=0.022; MGRVI: H=9.46, p=0.024; VARI: H=9.15, p=0.027). Given the small number of biological replicates, these omnibus *p*-values should be interpreted cautiously and primarily as evidence of an overall treatment effect rather than as evidence for specific pairwise differences. The vessel-level means showed a hump-shaped pattern, with the highest values observed at 7.5 g L^−1^ and progressively lower values at 15 and 30 g L^−1^: for ExG, from 90.93±1.64 at 0 g L^−1^ to 92.11±4.26 at 7.5 g L^−1^, 81.98±3.30 at 15 g L^−1^, and 67.61±3.97 at 30 g L^−1^; GLI showed the same pattern (0.2906±0.0130, 0.3411±0.0150, 0.3006±0.0087, and 0.2673±0.0088 at 0, 7.5, 15 and 30 g L^−1^, respectively). Individual vessel-level values underlying these treatment means are reported in Table S8 (Supplementary Material).

Given the limited number of biological replicates, pairwise post-hoc comparisons were not pursued at the vessel level (see Section 4.7).

The concentration-dependent variation in shoot greenness documented above was reflected in the concentration-specific Youden-optimal thresholds. The global thresholds were θ=33.0 for ExG and θ=0.0658 for GLI. Concentration-specific thresholds differed from the global values particularly at 7.5 g/L (ExG: θ=42.0; GLI: θ=0.077), consistent with the higher greenness observed at this concentration, while at 0 and 30 g/L both indices required lower cut-off values (ExG: θ=26.0; GLI: θ=0.039 and θ=0.050 respectively), reflecting the reduced shoot greenness at these extremes (Table 2).

### 2.4. Benchmarking of RGB-Derived Projected Shoot Cluster Area Against the Point-Cloud Reference

#### 2.4.1. Single Global Threshold

**Figure 6:**
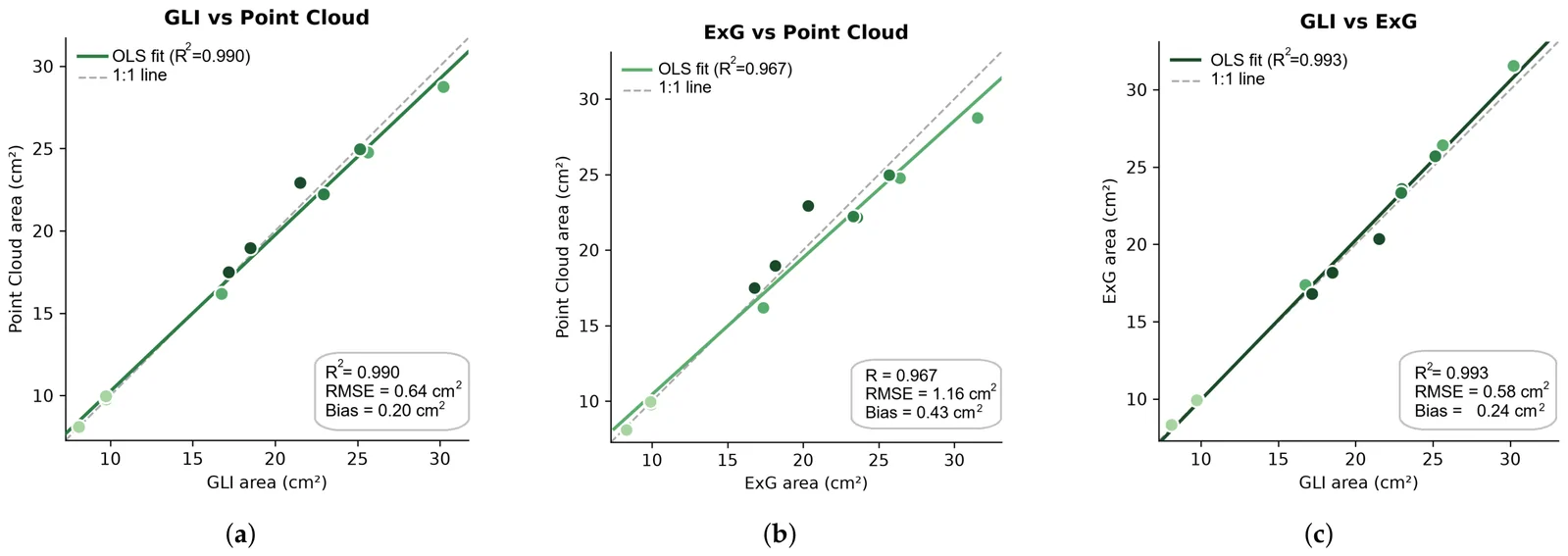
Linear regression between vessel-level projected shoot cluster area estimates (n=12 vessels) for three method comparisons: (**a**) GLI vs. Point Cloud; (**b**) ExG vs. Point Cloud; and (**c**) GLI vs. ExG. Points are coloured by sucrose concentration (0–30 g L^−1^, light to dark green). Solid line: ordinary least squares (OLS) regression fit; grey dashed line: 1:1 identity line.

Linear regression analysis at the vessel level (n=12) indicated a strong linear association between RGB-derived and point-cloud-derived shoot area estimates for both GLI and ExG. For GLI, the relationship showed $R^{2}=0.990$ and RMSE =0.64 cm^2^, whereas ExG showed $R^{2}=0.967$ and RMSE =1.16 cm^2^ (Figure 6; Table S3 in Supplementary Material). Bland–Altman analysis was used to complement the regression analysis by quantifying agreement and systematic bias. Regression RMSE is the root mean squared residual (divisor n=12) of an ordinary least squares fit of point-cloud area on RGB-derived area. For comparison, Table S3 also reports RMSE_BA_ and MAE, computed directly on the unadjusted paired differences (Section 4.7). RMSE_BA_ is necessarily at least as large as the regression RMSE, because the OLS fit minimises squared residuals and the identity line is one admissible line; no such constraint applies to MAE (e.g., ExG, single threshold: MAE =1.12 vs. regression RMSE =1.16 cm^2^). The mean bias was +0.20 cm^2^ for GLI and +0.43 cm^2^ for ExG (RGB − Point Cloud), indicating a small systematic overestimation of projected shoot cluster area by the RGB-derived methods relative to the point-cloud reference.

IoU analysis (see Table S4 in Supplementary Material) showed strong geometric agreement among all three method pairs, with every one of the 12 vessels exceeding the IoU =0.8 threshold. The highest agreement was observed between the two RGB-based methods (ExG vs. GLI: mean IoU =0.957±0.004, mean ± SEM; range 0.933–0.975), followed by GLI vs. Point Cloud (mean IoU =0.908±0.004; range 0.889–0.929) and ExG vs. Point Cloud (mean IoU =0.888±0.007; range 0.848–0.921). The minimum IoU was observed for each comparison in different vessels all belonging to the highest sucrose treatment (30 g/L), consistent with the greater variability in shoot appearance observed at this concentration (Section 2.3).

#### 2.4.2. Concentration-Specific Thresholds

Repeating the analysis using concentration-specific Youden-optimal thresholds instead of the single global threshold improved the in-sample regression fit for both indices (see Table S3 in Supplementary Material). For GLI, $R^{2}$ increased from 0.990 to 0.998, with RMSE decreasing from 0.64 to 0.28 cm^2^. For ExG, the improvement was similarly pronounced: $R^{2}$ increased from 0.967 to 0.996, with RMSE decreasing from 1.16 to 0.39 cm^2^.

This improvement in linear agreement was accompanied by a divergent change in systematic bias between the two indices: for GLI, the bias increased in magnitude (from +0.20 to +0.44 cm^2^), whereas for ExG it decreased (from +0.43 to +0.24 cm^2^). In contrast, direct paired errors decreased for both indices (GLI: RMSE_BA_ from 0.76 to 0.57 cm^2^ and MAE from 0.59 to 0.50 cm^2^; ExG: RMSE_BA_ from 1.40 to 0.55 cm^2^ and MAE from 1.12 to 0.51 cm^2^), even though the mean bias increased for GLI; concentration-specific thresholds therefore reduced the typical per-vessel error without reducing the mean signed difference for GLI (Table S3). The corresponding regression plots for both thresholding strategies are provided as Supplementary Figure S4.

In contrast, mean IoU values remained essentially unchanged when using concentration-specific thresholds (GLI vs. Point Cloud: 0.905 vs. 0.908 for the single threshold; ExG vs. Point Cloud: 0.898 vs. 0.888), indicating that the improvement in regression agreement was not systematically mirrored by an improvement in geometric (per-vessel) agreement. The comparison highlights that numerical agreement in total area does not necessarily imply improved spatial segmentation, supporting the use of complementary regression and overlap metrics. Direct comparison between the two thresholding strategies across all 12 vessels (Figure S6 in Supplementary Material) showed only minor differences in estimated shoot cluster area on average (mean Δ area =−0.20±0.41 cm^2^, mean ± SEM, for ExG; +0.24±0.24 cm^2^ for GLI) and in IoU relative to the point cloud (Δ IoU =+0.010±0.006 for ExG; −0.003±0.004 for GLI), although clear treatment-specific patterns emerged: at 7.5 g/L, concentration-specific thresholds systematically reduced estimated area (mean Δ=−1.90 cm^2^ for ExG, −0.84 cm^2^ for GLI) while improving agreement with the point cloud (mean Δ IoU =+0.034 for ExG, +0.014 for GLI); at 0 g/L, the opposite pattern was observed, with concentration-specific thresholds increasing estimated area (mean Δ=+0.51 cm^2^ for ExG, +0.85 cm^2^ for GLI) but slightly reducing IoU (mean Δ=−0.015 for ExG, −0.023 for GLI). Overall, these results indicate that concentration-specific thresholds provide a substantial improvement in regression-based (whole-dataset) agreement, particularly at 7.5 g/L, without a corresponding systematic improvement in per-vessel geometric agreement, and that the single global threshold remains a robust and considerably simpler alternative for automated shoot segmentation across the tested range of sucrose concentrations.

#### 2.4.3. External Validation

When applied without recalibration, following the procedure described in Section 4.8, to a separate set of blackberry vessel (*n* = 6; 10 and 15 explants, 30 days subculture) from a previous study (Regni et al. [30]), the global thresholds calibrated in Section 4.6 yielded RGB-derived projected shoot cluster areas in good agreement with the point cloud reference (Table S5 and Figure S5. GLI achieved $R^{2}=0.942$, RMSE $=0.32\;{\mathrm{cm}}^{2}$, and a mean bias of $-0.35\;{\mathrm{cm}}^{2}$ (RGB − Point Cloud); ExG yielded $R^{2}=0.909$, RMSE $=0.41\;{\mathrm{cm}}^{2}$, and bias $=-0.36\;{\mathrm{cm}}^{2}$. Direct paired errors on the unadjusted differences were RMSE_BA_=0.49 and MAE =0.45 cm^2^ for GLI, and RMSE_BA_=0.57 and MAE =0.54 cm^2^ for ExG; the regression RMSE values above are defined as in Section 2.4.1.

Although based on a limited number of vessels, these results provide preliminary evidence of threshold transferability beyond the dataset used for calibration, including across different SfM processing pipelines.

Against the manually annotated masks (Table S10), GLI reached a mean IoU of 0.908 (range 0.897–0.921) and ExG of 0.861 (0.838–0.888); mean sensitivity was 0.949 (0.933–0.963) for GLI and 0.921 (0.896–0.957) for ExG, and mean specificity 0.992 and 0.988, respectively. GLI outperformed ExG in IoU and sensitivity in all six vessels. The mean IoU of GLI was identical to that obtained against the point-cloud reference in the calibration vessels (0.908), whereas that of ExG was lower (0.861 vs. 0.888). Net area bias against the manual masks was close to zero (−0.05 and −0.06 cm^2^ for GLI and ExG, i.e., −0.7% and −0.9% of the manual area); the mean absolute bias was 0.13 and 0.20 cm^2^ (1.8% and 2.8%), with a maximum of 2.7% and 5.7%, respectively (Table S11). However, this concealed larger compensating errors: false-positive plus false-negative areas averaged 9.6% (GLI) and 14.8% (ExG) of the manual area. The manual masks were on average 0.30 cm^2^ (4.1%) smaller than the point-cloud areas of Table S5, which accounts for most of the negative bias of the RGB areas against the point cloud reported above.

## 3. Discussion

The Structure-from-Motion pipeline adopted here served two complementary functions. First, it produced a metrically-corrected orthophoto for each vessel, free from perspective distortion, from which the RGB vegetation indices used for shoot segmentation were computed, enabling projected shoot cluster area to be expressed in absolute physical units (cm^2^). Second, the same 3D reconstruction provided a geometrically explicit point cloud used exclusively as a benchmark reference for the RGB-derived shoot cluster area estimates (Section 2.4). The pixel-level ROC analysis should be interpreted as a calibration and class-separability analysis rather than as an independent segmentation validation, because the reference SC masks were derived from colour-filtered point clouds reconstructed from the same image set. The same caveat applies to the vessel-level agreement metrics reported in Section 2.4 ($R^{2}$, RMSE, IoU): because the point cloud is itself derived from the same RGB image set via colour-based filtering, these metrics quantify consistency between two processing pipelines applied to the same source data, rather than agreement against a fully independent ground truth.

The relative performance of GLI and ExG observed here contrasts with the mixed and at times contradictory rankings reported for these same indices under open-field and UAV-based acquisition conditions. Kazemi and Ghanbari Parmehr [22] found MGRVI to correlate best with multispectral NDVI in rice monitoring ($R^{2}=0.72$–0.73), while GLI performed poorly under the same UAV field conditions ($R^{2}=0.46$); Barbosa et al. [26] similarly found MPRI and MGRVI to outperform GLI and ExG for grass/soil discrimination under variable UAV lighting, with GLI showing an inverse response pattern under shadowed conditions; Santos et al. [27] found that the best-performing index varied by dataset across four RGB acquisition systems, concluding that no single index reliably segments plants from background under heterogeneous conditions; and contrasting rankings were reported by Bassine et al. [23], who found ExG–ExR most stable for UAV-based vegetation recognition, and by Eng et al. [25], who instead judged GLI the best-performing index on urban and forested UAV imagery.

The comparatively strong and stable performance of GLI and ExG here (AUC-ROC =0.991 and 0.988, respectively) may partly reflect the controlled acquisition conditions: the spatially homogeneous background and fixed, short imaging distance minimise the illumination and background heterogeneity that may underlie the contrasting rankings reported across studies.

The comparatively strong and stable performance of GLI and ExG in the present study (AUC-ROC =0.991 and 0.988, respectively) may partly reflect the relatively controlled acquisition conditions: the contrasting performance reported across studies may partly arise from differences in illumination and background heterogeneity, whereas the spatially homogeneous background and fixed, short imaging distance used here minimise these sources of variability, allowing both indices to achieve high discriminative performance.

The vessels’ transparent, gas-permeable plastic housing likely also contributes to this favourable performance: unlike the opaque soil or canopy backgrounds typical of field and UAV imaging, the translucent vessel walls allow diffuse light transmission and largely eliminate cast shadows on the shoot clusters, further reducing a major source of within-class spectral variability affecting ExG- and GLI-type indices under outdoor conditions. This instability across field studies reinforces the rationale for the empirical, condition-specific threshold calibration adopted here (ROC/Youden-based), rather than assuming that index rankings established in field literature transfer directly to close-range, controlled-background conditions. It should be noted that the close numerical agreement between GLI and ExG is partly expected given their algebraic relationship: GLI is a normalised form of ExG, sharing the same numerator (2G−R−B; Equations (1) and (2)), so that part of their high mutual correlation reflects this mathematical structure rather than independent confirmation of segmentation performance.

Beyond field- and canopy-scale applications, RGB-derived greenness metrics have also been applied to fine-scale, controlled-background phenotyping, for example as a near-surface reference for NDVI phenology [28] and to quantify seedling greenness during de-etiolation [29]. Both share with the present study computational simplicity and reliance on standard RGB imagery, but neither provided a geometric benchmark for derived area estimates. The present study extends this line of work by benchmarking RGB-derived projected shoot cluster area against the SfM-based point cloud and by empirically calibrating a classification threshold rather than relying on a fixed rule, which may improve transferability across related acquisition conditions and culture set-ups.

Within in vitro culture, earlier image-based approaches to growth monitoring relied on 2D imaging without geometric benchmarking [37,38], in line with the continued reliance on planar, non-volumetric approaches noted more broadly in plant phenotyping [39]. The present study extends these approaches by benchmarking RGB-derived projected shoot cluster area against a point-cloud reference in a micropropagation context ($R^{2}=0.990$ for GLI, $R^{2}=0.967$ for ExG, single global threshold), which, under the tested conditions, gives quantitative grounding to methods that had previously relied on visual or colour-based criteria alone.

The reliability of this geometric benchmark depends on the accuracy of the underlying acquisition hardware. The viDoc RTK Rover coupled with a LiDAR-equipped smartphone has been independently validated for sub-centimetre geometric accuracy, including directly against Terrestrial Laser Scanning in an arboreal context [40,41,42], supporting the reliability of the point cloud reference used here, although these benchmark studies do not constitute an independent accuracy assessment of the present vessel-level point clouds. The small residual disagreement between RGB- and point cloud-derived shoot area (IoU = 0.888–0.957, all vessels above the IoU = 0.8 agreement threshold) may therefore be more plausibly attributable to the colour-based segmentation and thresholding process than to geometric error in the acquisition system. Consistently with this interpretation, the vessels showing comparatively lower agreement were concentrated at the highest sucrose concentration (30 g L^−1^), where greater variability in shoot appearance (Section 2.3) likely increased the difficulty of the colour-based classification task.

Regni et al. [30] and Regni et al. [31] represent the closest methodological precedent to the present work, sharing the same acquisition hardware and SfM pipeline (see Introduction) but differing in species and analytical depth: planar shoot-covered area in blackberry and blueberry [30], versus fully volumetric shoot-level descriptors (height, volume, shape indices) in kiwifruit, which also revealed that individual-shoot segmentation becomes unreliable at high explant densities due to canopy overlap [31].

In both studies, the reconstructed point cloud served as the primary source of morphometric ground truth. The present work inverts this role, using the point cloud exclusively as a geometric benchmark against which a simpler 2D vegetation-index-based segmentation method is benchmarked. The resulting geometric benchmarking supports the quantitative performance of the RGB-based approach ($R^{2}=0.990$, RMSE =0.64 cm^2^ for GLI; $R^{2}=0.967$, RMSE =1.16 cm^2^ for ExG, single global threshold), indicating that, once the classification threshold has been empirically calibrated, an RGB-based segmentation approach applied to SfM-derived orthophotos can reproduce shoot cluster area estimates obtained from the corresponding 3D photogrammetric reconstruction with only a small, consistent overestimation (bias =+0.20 cm^2^ for GLI, +0.43 cm^2^ for ExG). This positions RGB-based area extraction from SfM-derived orthophotos as a simple, calibrated alternative to colour-filtering of the point cloud for estimating projected shoot cluster area; it does not avoid 3D reconstruction, which remains necessary to generate the orthophotos and the reference. Moreover, because the classification operates on a single top-down projection of each vessel, it cannot resolve overlap or mutual occlusion between adjacent shoots, nor their vertical development; both remain accessible only through the 3D point cloud, and the volumetric approach of Regni et al. [31] is required when finer morphological descriptors (height, volume, shape indices) are needed.

Automated phenotyping platforms developed specifically for in vitro culture (e.g., [16]) rely instead on laser distance sensors, spectrometry, and thermal imaging to characterise culture performance in situ. Such platforms offer richer, multi-dimensional phenotypic data, but at the cost of specialised instrumentation unlikely to be broadly accessible to micropropagation laboratories. The RGB-only approach evaluated here trades this additional sensor richness for simplicity and relatively low cost, while still providing an area estimate benchmarked against a geometrically explicit point-cloud reference.

The use of concentration-specific Youden-optimal thresholds yielded an improvement in regression performance relative to the single global threshold (GLI: $R^{2}$ from 0.990 to 0.998, RMSE from 0.64 to 0.28 cm^2^; ExG: $R^{2}$ from 0.967 to 0.996, RMSE from 1.16 to 0.39 cm^2^), without a corresponding systematic improvement in per-vessel geometric agreement (mean IoU essentially unchanged: GLI 0.905 vs. 0.908; ExG 0.898 vs. 0.888). This dissociation between regression- and IoU-based agreement metrics suggests that concentration-specific thresholds primarily reduce systematic, treatment-level area biases, most pronounced at 7.5 g L^−1^, where higher shoot greenness shifts the optimal cut-off well above the global value, rather than improving the spatial overlap of individual vessel segmentations. In practical terms, the single global threshold therefore remains a robust and considerably simpler choice for routine applications, provided that whole-dataset comparisons rather than per-treatment estimates are the primary analytical target. This supports its use without treatment-specific recalibration across the sucrose concentrations tested here. Concentration-specific thresholds may be useful when treatment-specific calibration is explicitly intended, although their performance should be independently validated before being recommended for quantitative comparisons across treatments.

The stability of classification performance across sucrose treatments (AUC-ROC ranging from 0.978 to 0.994 for ExG and from 0.987 to 0.995 for GLI), together with the leave-one-vessel-out cross-validation and the external validation results reported below, suggests that the empirically calibrated thresholds are not strongly overfitted to the specific vessels used for calibration. Applying the global thresholds without recalibration to a separate blackberry dataset from a previous study acquired with a similar procedure (Regni et al. [30]) yielded $R^{2}=0.942$, RMSE =0.32 cm^2^ for GLI and $R^{2}=0.909$, RMSE =0.41 cm^2^ for ExG. The systematic bias was comparable in magnitude between the calibration and validation datasets, although opposite in direction (RGB slightly overestimating the point cloud in the main dataset, and slightly underestimating it in the separate validation set; against manually annotated masks, however, the bias in the separate set was close to zero (−0.05 cm^2^ for GLI), indicating that the negative bias relative to the point cloud largely reflects differences between the two references rather than the indices). The small magnitude of the bias relative to the limits of agreement in both cases, together with the absence of a consistent trend in its sign, is consistent with sampling variability around a near-zero true bias rather than a directional method artefact. Although the validation sample is small (n = 6 vessels) and limited to a single cultivar and subculture stage, this result provides a first, though encouraging, indication of threshold generalisability across different SfM processing pipelines. Given this limited scope, the external validation reported here should be interpreted as a qualitative proof-of-concept for threshold transferability rather than a quantitative estimate of its robustness. Validation on larger and more diverse datasets, spanning multiple cultivars, acquisition conditions, and subculture stages, will be needed to consolidate these preliminary findings, potentially extending to other in vitro-propagated soft fruit species, such as blueberry (*Vaccinium* spp.), for which micropropagation protocols are well established [44]. Although all acquisitions used automatic exposure and white balance under natural indoor lighting, illumination variability across vessels and acquisition sessions was not formally quantified (e.g., via lux measurements), and represents an uncontrolled source of variability not accounted for in the present analysis. Because RGB indices are derived from smartphone image data, threshold transferability may be sensitive to illumination, camera settings and image-processing conditions, and the external dataset used comparable hardware and a related set-up; validation under different conditions is therefore required.

As a final practical consideration, because the RGB classification step relies exclusively on pixel-level colour values rather than on any metrically-corrected or georeferenced product, it is not intrinsically tied to the orthophoto/SfM pipeline required to express area in absolute units. Applied directly to an ordinary, non-orthorectified image acquired under a standardised set-up (fixed top-down position and camera-to-vessel distance), the same index-and-threshold classification could yield purely relative phenotypic descriptors (such as the fraction of vessel area occupied by shoot tissue, shoot-to-vessel area ratios, or relative shoot density) without any need for ground control targets, RTK positioning, or photogrammetric reconstruction. If confirmed by dedicated validation, this could lower the barrier to routine adoption in laboratories without access to photogrammetric equipment, and the framework evaluated here could serve as a starting point for the development of fully automated, low-cost shoot monitoring systems compatible with standard laboratory imaging set-ups.

Future studies will specifically investigate the reproducibility of this analysis when applied to single, non-orthorectified images, explicitly accounting for standardised illumination conditions to minimise exposure- and lighting-related variability in the computed indices.

## 4. Materials and Methods

### 4.1. Plant Material and In Vitro Culture Conditions

The blackberry (*Rubus* spp cv. ’Thornfree’) explants were derived from stock plant material maintained under aseptic conditions at the Micropropagation and In Vitro Biotechnology Laboratory, Research Unit “Tree Science,” Department of Agricultural, Food and Environmental Sciences, University of Perugia (Italy). The medium comprised half-strength Murashige and Skoog salts [45], supplemented with different sucrose concentrations (0, 7.5, 15, and 30 g L^−1^), indole-3-butyric acid (IBA, 0.1 mg L^−1^), 6-benzylaminopurine (BAP, 0.4 mg L^−1^), and agar (8 g L^−1^) as gelling agent; medium pH was adjusted to 5.7 prior to sterilization. Cultures were established in transparent, autoclavable polypropylene ventilated vessels (8 cm height, 9.7 cm basal diameter, 565 mL nominal volume; Sac O2, Deinze, Belgium) containing 100 mL of the culture medium described above. Vessels and medium were autoclaved (115 °C, 20 min) prior to culture initiation. Explant material consisted of uninodal segments 1.2–1.5 cm in length and bearing 3–4 fully expanded leaves. Fifteen explants per vessel were used. The explants were then allocated in a controlled-environment growth chamber under a constant temperature of 22±2 °C, a 16-h photoperiod, and a photosynthetic photon flux density of 40 µE m^−2^ s^−1^, for a culture period of 30 days.

### 4.2. Experimental Design

The trial was conducted using the four sucrose regimes outlined above (0, 7.5, 15, and 30 g L^−1^), with three independent vessels, each containing 15 shoots. Explants were arranged within each culture vessel according to the standardized spatial layout described in Brigante et al. [32].

Non-invasive three-dimensional acquisitions were performed throughout the experiment. Vessel lids were removed prior to image acquisition to avoid reflections and optical distortion from the transparent lid surface, and were replaced immediately afterwards to minimise contamination risk and maintain culture conditions. The present study is based exclusively on the acquisitions collected at the end of the 30-day subculture period, which were used for all RGB vegetation index and shoot area analyses.

**Figure 7:**
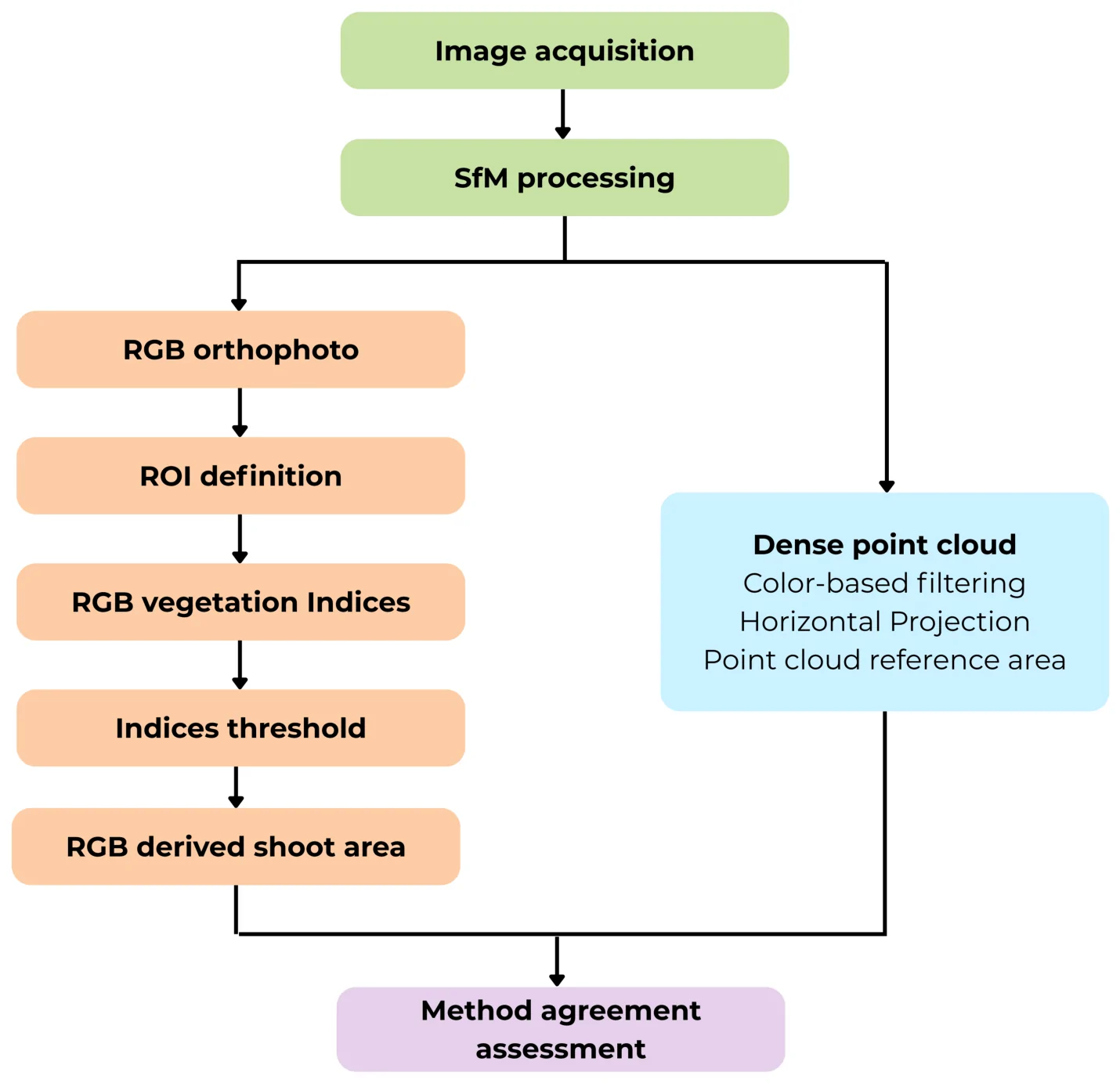
Methodological workflow of the proposed approach. From the same set of acquired images, Structure-from-Motion (SfM) processing generates both a two-dimensional RGB orthophoto, used to compute RGB vegetation indices, define classification thresholds, and derive the RGB-based shoot cluster area, and a three-dimensional dense point cloud, used exclusively as a geometric reference through colour-based filtering, horizontal projection, and area extraction. The two derived area estimates are then compared in the final method agreement assessment.

The methodological workflow of the proposed approach is reported in Figure 7.

### 4.3. Image Acquisition and Photogrammetric Processing

For each vessel, a set of about 115 photographs was acquired using an Apple iPhone 15 Pro Max coupled with a viDoc RTK rover, following the same acquisition protocol described in Regni et al. [30,31]. Images were acquired using the default automatic exposure and white balance settings of the smartphone camera via the Pix4Dcatch application. The image acquisition process required only a few minutes per vessel: images were captured at a working distance of approximately 15 cm from the vessel, under natural lighting conditions, with image overlap (target 90%) managed automatically by the Pix4Dcatch application (Pix4D SA, Prilly, Switzerland). Each acquisition was performed with the culture vessel placed on a custom cardboard base printed with five ground control targets surrounding the vessel, enabling scale definition of the resulting 3D reconstructions. The same set of images was used both to generate the two-dimensional RGB orthophoto and to reconstruct the three-dimensional point cloud for each vessel.

**Figure 8:**
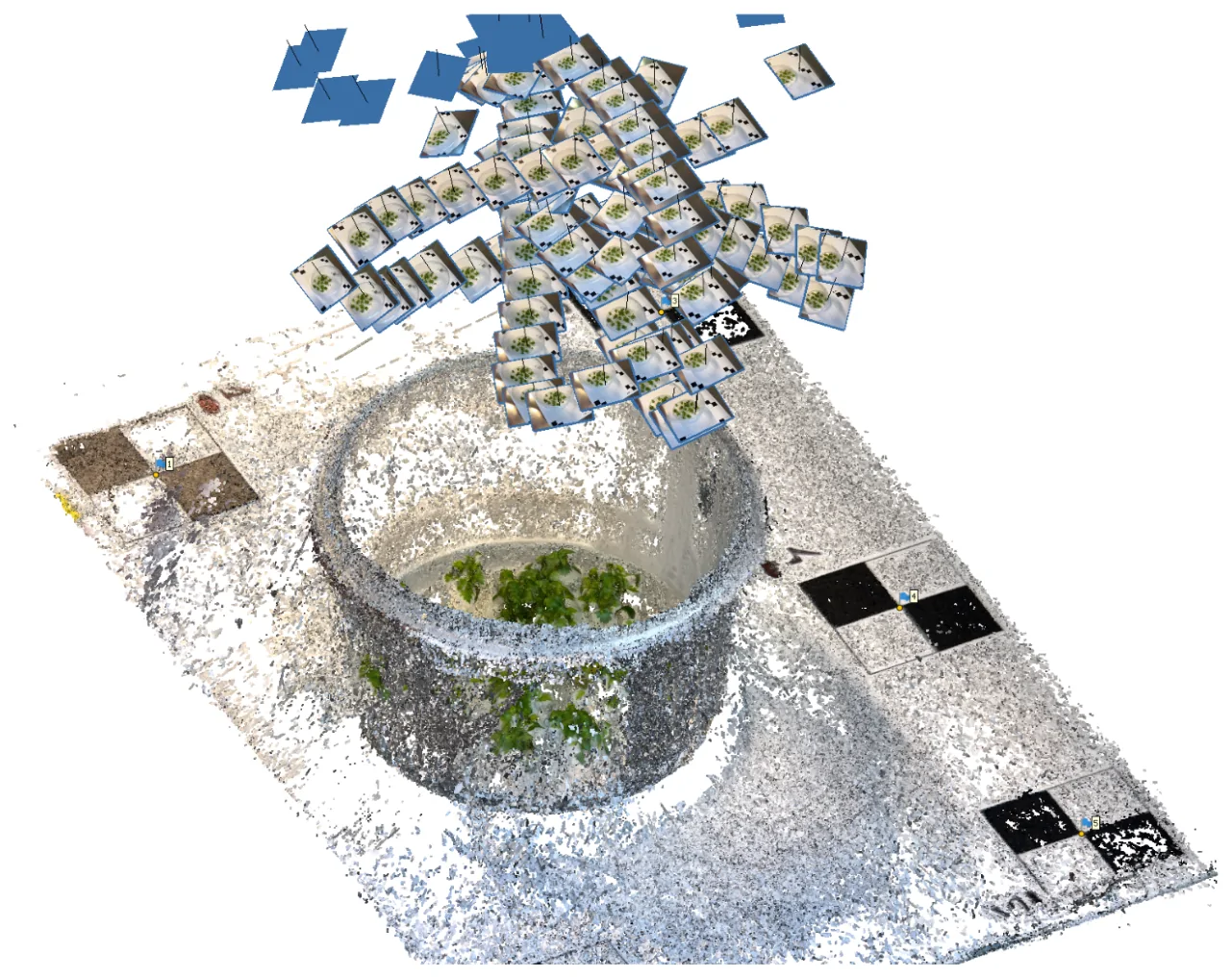
Photogrammetric reconstruction of a representative vessel in Agisoft Metashape, showing the reconstructed dense point cloud and the estimated positions of the approximately 115 camera stations used for image acquisition, and the ground control targets printed on the supporting base. The five printed ground control targets (chekerboards visible on the base) provided the scale reference for the reconstruction (Section 4.3).

**Figure 9:**
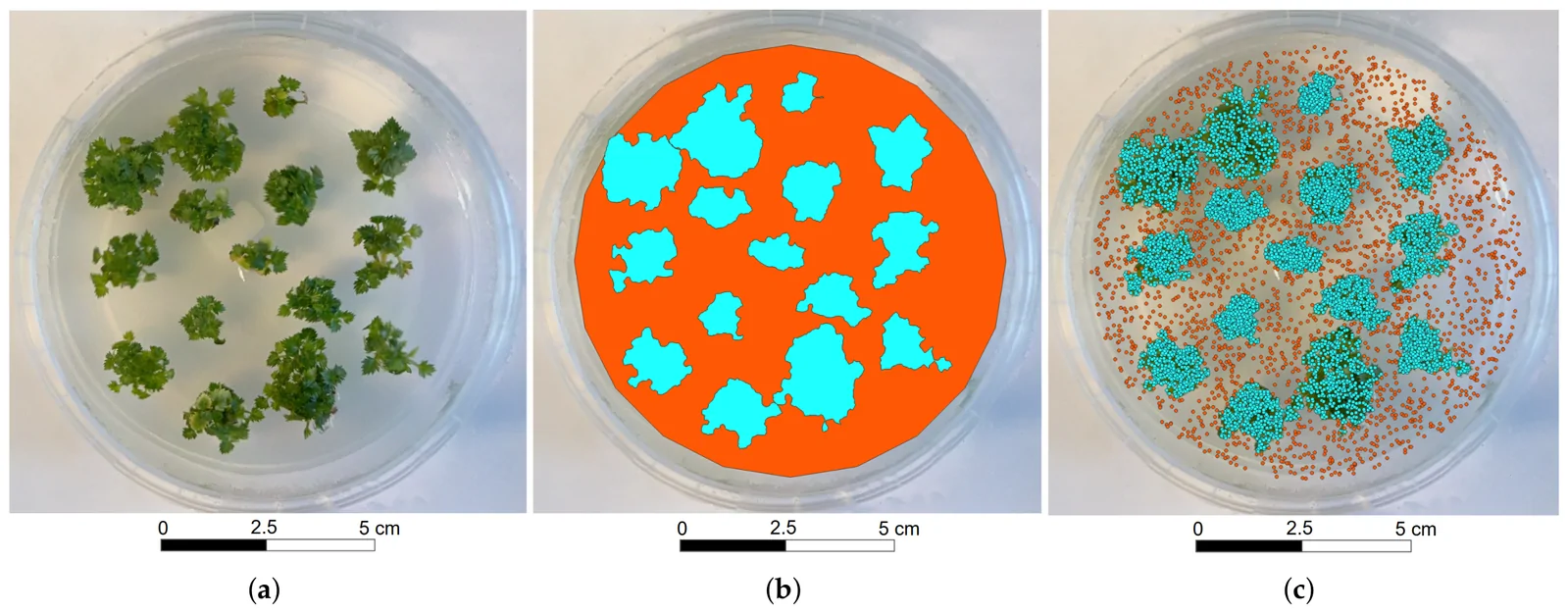
Definition of the Shoot cluster (SC) and Background (BG) regions of interest (ROIs) for a representative vessel (TF-15C, 15 g L^−1^ sucrose). (**a**) Orthophoto. (**b**) Final Shoot cluster (cyan) and Background (orange) ROIs. (**c**) Points classified as SC and BG shown in the same colour scheme.

Both the orthophoto and the dense point cloud were generated in Agisoft Metashape Professional (version 1.7.4, Agisoft LLC, St. Petersburg, Russia), following the same processing workflow adopted in Regni et al. [31] (Figure 8). Images were aligned using a marker accuracy setting of 2 mm, with key point and tie point limits set to 40,000 and 4000 respectively. A dense point cloud was subsequently built at high quality with mild depth filtering, from which both the orthomosaic (used for RGB vegetation index computation; see Section 4.5) and the 3D point cloud (used exclusively as a reference for area benchmarking; see Section 2.4) were derived. Rather than relying on estimated camera positions, the five ground control targets placed around each vessel were used directly within Metashape to define the scale of the model, yielding a sub-millimetre mean reprojection error on the ground control points positions. The resulting dense point clouds likewise showed a sub-millimetre mean point spacing and were used to extract the orthophoto (Figure 9a) for each vessel with a geometric resolution of $6\times 10^{-5}$ m.

### 4.4. Definition of “Shoot Cluster” (SC) and “Background” (BG) Regions of Interest (ROI)

The dense point cloud was used both as a geometric benchmark (Section 2.4) and to generate the two regions of interest (ROIs) considered in this study: the “Shoot cluster” (SC) and “Background” (BG) classes. Shoot points were extracted from the dense point cloud in Cyclone 3DR (Leica Geosystems, Heerbrugg, Switzerland) using the “Real Colors” colour-extraction tool. Reference colour values were obtained by manually sampling a set of points on the vessel body, base, and other non-plant surfaces, all characterised by a pale grey tone, and the Tolerance slider was left at its default (intermediate) position between the “Strict” and “Tolerant” limits. Point-cloud points falling within the resulting colour tolerance window were automatically identified and discarded, leaving only the green shoot-tissue points. Isolated points were then removed with the “Remove noise” filter, with the Intensity slider set to 80 and the valid points retained. The filtered shoot cloud was projected onto a horizontal plane, and the planar outlines were extracted as vector contours, yielding the individual SC ROIs; the area enclosed by these contours was measured in Cyclone 3DR to obtain the projected shoot cluster area of each vessel. This procedure followed the colour-segmentation workflow described in Regni et al. [30]. The BG class was obtained in QGIS (version 3.34) by subtracting the SC polygons from a circular area matching the vessel diameter, yielding one BG ROI per vessel. Each of the twelve vessels was thus described by 15 SC ROIs (17 in TF-30A, where two small detached leaflets were digitised as separate polygons; 182 SC ROIs in total) and 1 BG ROI (Figure 9b), used for all ROI-level analyses described in Section 4.5 and Section 4.7.

In addition to this ROI-level analysis, a set of sampling points was generated within each Shoot cluster and Background polygon in QGis (Figure 9c), enabling all analyses to be performed also at the pixel level.

Specifically, the sampling procedure, applied across all culture vessels, was performed separately for each polygon, following a class-specific allocation strategy (500 points per each SC polygon and 2000 points per BG polygon) to account for the variability of RGB vegetation index within the SC ROI, which is less homogeneous in terms of RGB values than the Background; this resulted in a substantially larger total sampling effort devoted to the SC class. This allocation was fixed per polygon rather than scaled to polygon area, and was applied to all twelve vessels. The nominal allocation (182 SC and 12 BG polygons) corresponds to 91,000 SC and 24,000 BG points; the final dataset (92,574 SC and 23,924 BG points) differs marginally from it, owing mainly to a higher sampling density in one vessel (TF-0A) and to minor vessel-specific adjustments, without effect on the calibrated thresholds.

### 4.5. RGB Vegetation Index Computation

**Figure 10:**
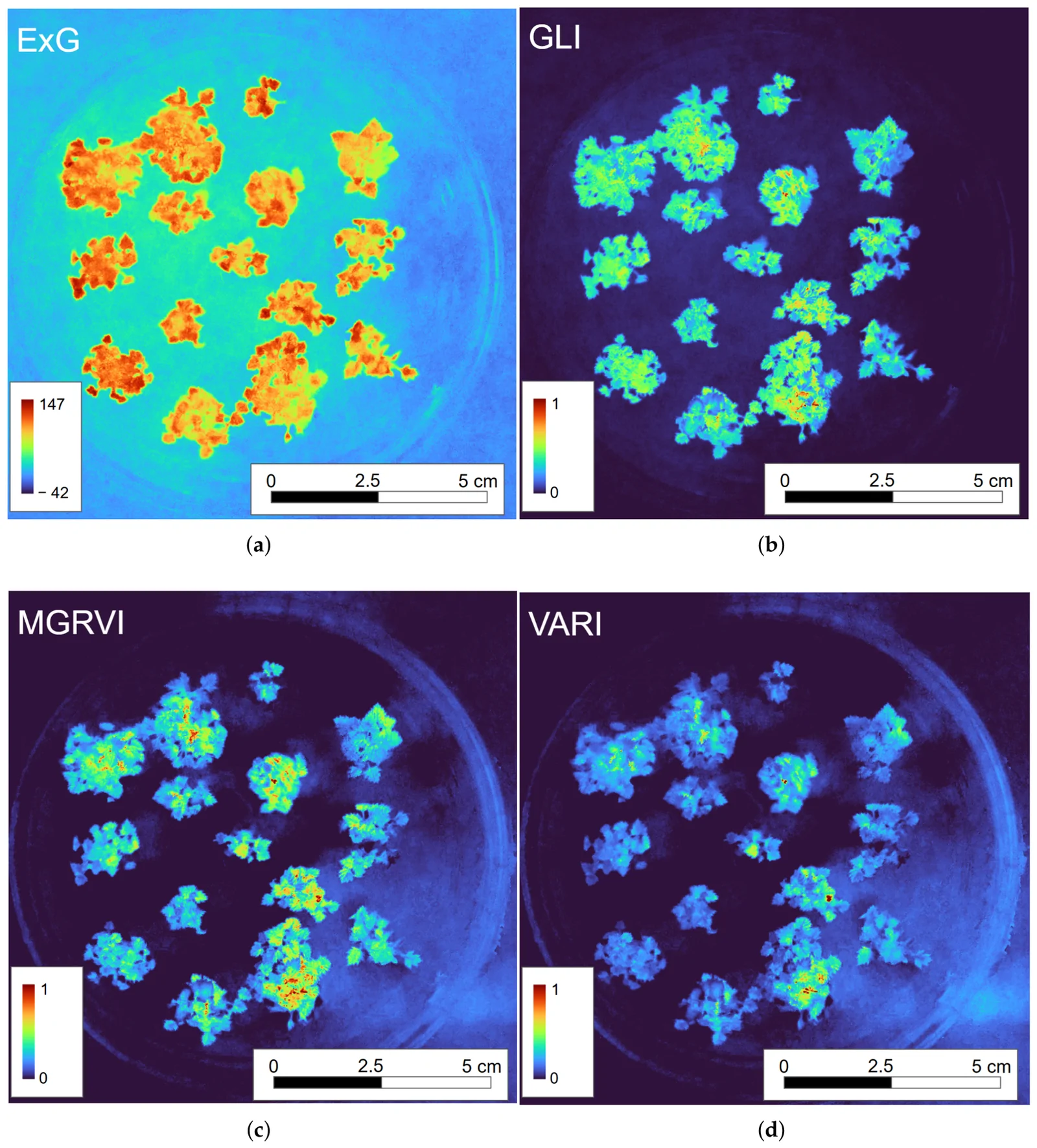
Maps of the four RGB vegetation indices computed for a representative vessel (TF-15C, 15 g L^−1^ sucrose): (**a**) Excess Green (ExG), (**b**) Green Leaf Index (GLI), (**c**) Modified Green Red Vegetation Index (MGRVI), and (**d**) Visible Atmospherically Resistant Index (VARI).

Four RGB-based vegetation indices were computed from each orthophoto in QGIS: Excess Green (ExG), Green Leaf Index (GLI), Modified Green Red Vegetation Index (MGRVI), and Visible Atmospherically Resistant Index (VARI) (Figure 10). For each pixel, the four indices were computed directly from the Red (*R*), Green (*G*), and Blue (*B*) digital number values extracted from the orthophoto (0–255 range), according to Equations (1)–(4):

(1) ExG=2G−R−B

(2) $$\mathrm{GLI}=\frac{2G-R-B}{2G+R+B}$$

(3) $$\mathrm{MGRVI}=\frac{G^{2}-R^{2}}{G^{2}+R^{2}}$$

(4) $$\mathrm{VARI}=\frac{G-R}{G+R-B}$$

The four indices were analysed at two complementary levels. At the ROI level, the SC and BG masks described in Section 4.4 were used to summarise each index within every polygon, computing descriptive statistics (mean, minimum, maximum, and standard deviation) of the pixel values contained in that polygon. At the pixel level, instead, the value of each index was retained for every individual sampling point within the SC and BG masks; descriptive statistics (mean, standard deviation, median, and interquartile range) were then computed on the resulting pixel-level distributions for each class, providing a complementary characterisation of within-class spectral variability at a finer spatial scale than the ROI-level summaries.

### 4.6. Classification Threshold Determination and Shoot Cluster Area Estimation

The classification threshold used to separate SC from BG pixels was determined via ROC/Youden-index optimisation (see Section 4.7 for the underlying statistical framework), applied both as a single global threshold (pooling pixels from all sucrose treatments) and as treatment-specific thresholds (computed separately for each of the four sucrose concentrations).

**Figure 11:**
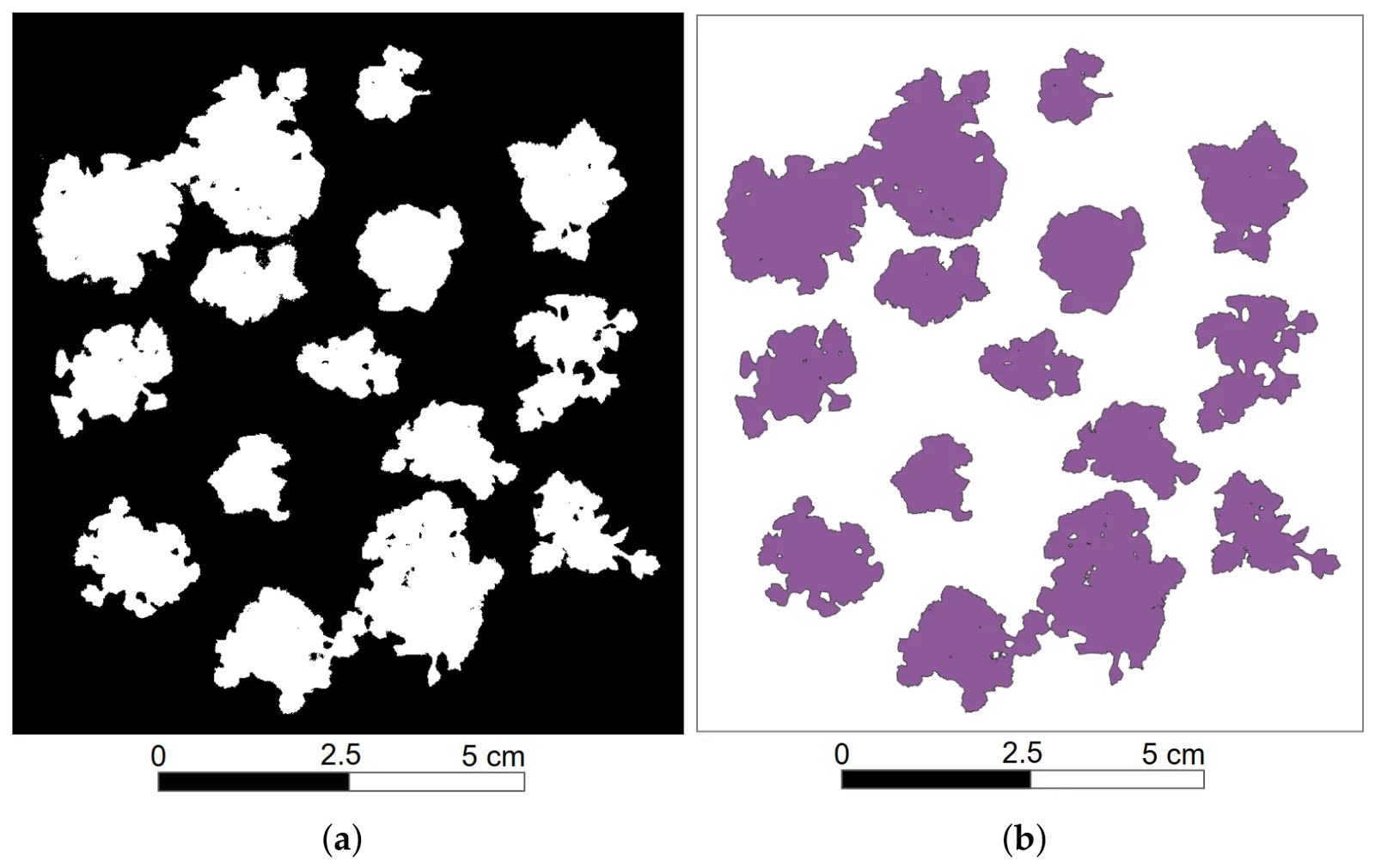
From vegetation index to RGB-derived projected shoot cluster area, for a representative vessel (TF-15C, 15 g L^−1^ sucrose). (**a**) Binary reclassification of the GLI map of vessel TF-15C (15 g L^−1^ sucrose) at the single global threshold (θ=0.0658), separating SC (white) from BG (black) pixels. (**b**) Vectorisation of the classified Shoot pixels into polygons, from which RGB-derived shoot area was computed.

For each vessel, the selected threshold was applied to each vegetation index to separate the SC and BG classes. The two indices for which the best class separability was obtained were then used to generate a binary Shoot cluster/Background mask, assigning pixels with digital number (DN) below the threshold to the BG class and those with values equal to or above the threshold to the SC class, obtaining a 1-bit raster with DN = 0 for BG class and DN = 1 for SC class (Figure 11a). The binary raster was subsequently vectorized in QGIS, and the area of the resulting SC polygons (Figure 11b) within each vessel boundary was calculated. This procedure was repeated independently using both the global and treatment-specific thresholds for the two selected indices (ExG and GLI), yielding one RGB-derived projected shoot cluster area estimate per vessel, per index, and per thresholding strategy.

The numerical values of both the global and treatment-specific thresholds, together with the associated classification performance metrics, are reported in Section 2.1.

The point cloud-derived reference area for each vessel (Section 4.3) provided a geometrically explicit reference against which the RGB-derived area estimates were benchmarked (Section 2.4). Agreement between the two methods was assessed via linear regression and Bland-Altman analysis, and their spatial overlap via the Intersection over Union (IoU) metric, for both the single global threshold and the treatment-specific thresholds.

### 4.7. Statistical Analysis

All analyses were performed in Python (Anaconda distribution, v.3.12), using pandas (2.3.3), NumPy (2.3.5), Matplotlib (3.10.6), SciPy (1.16.3), and scikit-learn (1.7.2).

Class separability at the pixel level was quantified using two complementary metrics:

- the Separability Ratio (SR), computed by Equation (5)
  (5) $$SR=\frac{|\mu_{\mathrm{Shoot}}-\mu_{\mathrm{Background}}|}{\sigma_{\mathrm{Shoot}}+\sigma_{\mathrm{Background}}}$$
  where μ and σ denote, respectively, the mean and standard deviation of a given index within each class; values of SR >1 indicate excellent separation;
- the area under the ROC curve (AUC−ROC), used to summarise the overall discriminative capacity of each index across the full range of possible classification thresholds.

Differences among sucrose treatments were assessed using the Kruskal-Wallis test (*H*; approximately $\chi^{2}$-distributed under the null hypothesis of no difference among treatments, with df=k−1 degrees of freedom, where *k* is the number of groups compared, i.e., df=3 for the four sucrose treatments considered here). To respect the true biological replication unit of the experiment, this test was performed at the vessel level: for each vessel, the mean of all SC ROI values for a given index was computed first, yielding three independent replicates per sucrose treatment (n=12 vessels total, n=3 per treatment). Pixel- and ROI-level distributions were retained for characterising class separability and calibrating classification thresholds (Section 2.1 and Section 4.6), but inferential comparisons among sucrose treatments were performed exclusively at the vessel level. Given the limited number of biological replicates (n=3 per treatment), pairwise post-hoc comparisons were not pursued at the vessel level because the resulting tests would have very limited statistical power. Treatment differences are therefore interpreted primarily based on the overall Kruskal–Wallis test, while the observed pairwise patterns are described qualitatively for context.

Classification thresholds were determined by Receiver Operating Characteristic (ROC) curve analysis, selecting the threshold that maximised the Youden index, defined by Equation (6):

(6) J=Sensitivity+Specificity−1

where Sensitivity and Specificity are defined, respectively, by Equations (7) and (8):

(7) $$\mathrm{Sensitivity}=\frac{TP}{TP+FN}$$

(8) $$\mathrm{Specificity}=\frac{TN}{TN+FP}$$

with TP (True Positive) and FN (False Negative) denoting the number of Shoot pixels correctly and incorrectly classified, respectively, and TN (True Negative) and FP (False Positive) denoting the number of Background pixels correctly and incorrectly classified, respectively.

It should be noted that pixel-level observations sampled within the same ROI are spatially autocorrelated and therefore not statistically independent. Accordingly, the large number of sampled pixels (n=116,498) should not be interpreted as an equivalent number of independent biological observations; pixel-level analyses were therefore used for descriptive class-separability assessment and threshold calibration, whereas inferential comparisons among sucrose treatments were performed at the vessel level.

Two threshold strategies were evaluated: a single global threshold, computed pooling pixels from all sucrose treatments, and treatment-specific thresholds, computed separately for each of the four sucrose concentrations.

Classification performance at the optimal global threshold was further summarised using:

- Accuracy, the overall proportion of correctly classified pixels,
  (9) $$\mathrm{Accuracy}=\frac{TP+TN}{TP+TN+FP+FN}$$
- Precision, the proportion of pixels classified as Shoot cluster that were correctly classified,
  (10) $$\mathrm{Precision}=\frac{TP}{TP+FP}$$
- the F1 score, the harmonic mean of Precision and Sensitivity,
  (11) $$F1=2\times \frac{\mathrm{Precision}\times \mathrm{Sensitivity}}{\mathrm{Precision}+\mathrm{Sensitivity}}$$

Agreement between RGB-derived and point cloud-derived shoot cluster area estimates was evaluated through linear regression and Bland-Altman analysis. Alongside the regression RMSE, direct paired errors were computed on the unadjusted differences *d* (RGB − Point Cloud) as RMSE_BA_$=\sqrt{\mathrm{mean}(d^{2})}$ and mean absolute error, MAE =mean(|d|). Spatial overlap between the segmentations was quantified with the Intersection over Union (IoU), defined as the area shared by the two segmentations divided by the area covered by at least one of them. Unless otherwise specified, IoU refers to the geometric overlap computed in QGIS between the complete vessel-level SC polygons obtained with each method (index-based classification and colour-filtered point cloud); its descriptive statistics (mean, standard deviation and percentile distribution) were computed separately for the single global threshold and for the treatment-specific thresholds (Section 2.4). A second, pixel-level measure, IoU_pixel_, was computed from the sampled points classified at pixel level, as TP/(TP+FP+FN); sensitivity (TP/(TP+FN)) and specificity (TN/(TN+FP)) were obtained from the same points. These pixel-level metrics were calculated for each vessel using the fixed global thresholds, and the variation among the three vessels of each sucrose treatment was summarised. Because the sampling allocation was fixed per class (500 points per SC polygon and 2000 per BG polygon), IoU_pixel_ depends on the sampling design and should not be read as an area ratio.

To assess whether the empirically calibrated thresholds generalise beyond the specific vessels used for calibration, a leave-one-vessel-out cross-validation (LOVO-CV) was performed: for each of the 12 vessels in turn, the Youden-optimal threshold was recalibrated using pixel data from the remaining 11 vessels, and classification performance was evaluated on the held-out vessel.

### 4.8. External Validation on a Separate Dataset

Although the point-cloud reference is geometrically distinct from the RGB segmentation, it is not fully independent at the data-source level because both products originate from the same image set and the point-cloud segmentation also incorporates colour information. The external validation on a separate dataset therefore provides a complementary test of threshold transferability.

To assess whether the calibrated single global thresholds generalise beyond the calibration dataset, ExG and GLI were computed on a separate set of blackberry (cv. ’Thornfree’) orthophotos from Regni et al. [30], corresponding to vessels with 10 and 15 explants after 30 days of subculture (*n* = 6 vessels), acquired with the same smartphone–viDoc RTK hardware and a comparable acquisition protocol, though processed with a different SfM software (Pix4Dmatic). The global Youden-optimal thresholds calibrated in Section 4.6 were applied without re-fitting, and the resulting RGB-derived shoot cluster areas were compared against the point cloud-derived reference areas reported in that study, using the same regression and agreement metrics described in Section 4.7.

To assess segmentation accuracy independently of the point-cloud reference, the shoot cluster of each of the six external vessels was manually digitised in QGIS on the RGB orthophoto alone, without access to the index-based classifications or to the point-cloud-derived masks: every element of the orthophoto visually distinguishable from the background (all leaves as well as small stems) was digitised as shoot tissue. The global thresholds were applied without recalibration (GLI ≥ 0.0658; ExG ≥ 33.0), and the resulting index masks were polygonised and compared with the manual masks. IoU, sensitivity and specificity were computed from the areas of the polygon intersections (TP: manual and index; FN: manual only; FP: index only), and the area error was expressed as RGB − manual.

## 5. Conclusions

This study developed and evaluated a reliable, empirically calibrated RGB-based method for automated shoot segmentation and area quantification in *Rubus* spp. micropropagation, using RGB orthophotos without recourse to multispectral or hyperspectral acquisition. Among the four RGB vegetation indices compared, GLI and ExG were the most reliable for separating shoot tissue from background at both the pixel and whole-vessel level. A single, empirically calibrated global threshold generalised across all four sucrose treatments, matching concentration-specific thresholds in terms of per-vessel geometric agreement while offering considerably greater simplicity, and thus supporting its use as the default choice for routine application.

Sucrose concentration significantly influenced index values in a non-monotonic, hump-shaped manner (p<0.03 for all four indices at the vessel level though based on a limited number of biological replicates, n=3 vessels per treatment, and therefore to be interpreted with due caution), peaking at 7.5 g L^−1^ rather than at the sucrose-free control, a pattern that did not compromise the stability of the segmentation method itself but that suggests a non-linear response of RGB-derived shoot greenness to sucrose concentration whose physiological basis remains to be established, warranting further investigation under controlled factorial designs.

RGB-derived segmentation from metrically corrected orthophotos reproduced the point-cloud-derived shoot area with high agreement under the tested conditions, with $R^{2}\;=\;0.990$ and RMSE =0.64 cm^2^ for GLI and $R^{2}\;=\;0.967$ and RMSE =1.16 cm^2^ for ExG using the single global threshold. These results support RGB-based segmentation of reconstructed orthophotos as a simple means of extracting projected shoot cluster area under the tested conditions; the 3D reconstruction was required to generate the orthophotos and the reference, and the demonstrated contribution is the area extraction, not the avoidance of reconstruction. Preliminary external validation on a separate blackberry dataset from a previous study, acquired with a similar procedure, yielded comparable agreement metrics and a mean IoU of 0.908 (GLI) and 0.861 (ExG) against manually annotated masks, providing an initial indication that the calibrated thresholds generalise beyond the specific dataset and processing pipeline used for calibration.

Overall, the single global GLI or ExG threshold emerges as a simple strategy for projected shoot cluster area extraction from reconstructed orthophotos under the tested conditions. Application to single, standardised images remains a proposed use requiring validation, as does broader applicability across cultivars, illumination conditions, vessels, acquisition geometries and developmental stages. The link between these image-derived measurements and shoot development, propagation performance or culture optimisation was not tested here and remains to be established.

Future work should assess the transferability of the calibrated thresholds to other in vitro-propagated species, culture vessels, and acquisition conditions; investigate the reproducibility of single-image analysis under standardised illumination conditions; explore the integration of the proposed workflow into fully automated, high-throughput phenotyping pipelines; and investigate whether the non-monotonic effect of sucrose concentration on shoot greenness observed here is consistent across cultivars and subculture stages.

## Figures and Tables

**Table 1:** AUC-ROC for ExG and GLI computed separately for each sucrose treatment at pixel level.

Sucrose (g/L)	*N* Sampled Points	ExG AUC	GLI AUC
0	30,000	0.9944	0.9950
7.5	28,500	0.9937	0.9942
15	28,500	0.9914	0.9924
30	29,498	0.9777	0.9869
Global	116,498	0.9879	0.9913

**Table 2:** Youden-optimal classification thresholds (θ) for ExG and GLI under the single global strategy (all sucrose concentrations pooled) and the concentration-specific strategy, with associated sensitivity (Se) and specificity (Sp).

		ExG			GLI		
Strategy	Sucrose	θ	Se	Sp	θ	Se	Sp
	(g L^−1^)						
Global	All	33.0	0.952	0.973	0.0658	0.957	0.981
Conc.-specific	0	26.0	0.976	0.988	0.0386	0.981	0.986
	7.5	42.0	0.964	0.984	0.0766	0.969	0.981
	15	37.0	0.954	0.980	0.0662	0.962	0.977
	30	26.0	0.934	0.969	0.0496	0.956	0.977

## Data Availability

The original contributions presented in this study are included in the article/Supplementary Material. Further inquiries can be directed to the corresponding author.

## Supplementary Materials

The following supporting information can be downloaded at: https://www.mdpi.com/article/10.3390/plants15213460/s1, Figure S1: Box-plots of pixel-level values for the four RGB vegetation indices (ExG, GLI, MGRVI, VARI) by class. Figure S2: Box-plots of pixel-level values for the four RGB vegetation indices (ExG, GLI, MGRVI, VARI) by class. Figure S3: Bland–Altman plots comparing vessel-level shoot area estimates obtained from RGB-based segmentation (GLI and ExG) with the corresponding point-cloud reference. Figure S4: Linear regression between RGB-based and point-cloud area estimates, comparing the single global threshold and concentration-specific thresholds for ExG and GLI. Figure S5: Linear regression between RGB-based and point-cloud-derived shoot area estimates for the external dataset. Figure S6: Differences between concentration-specific and single global threshold approaches. Table S1: Descriptive statistics (mean ± SD) of the four RGB vegetation indices for the Shoot cluster and Background classes at the ROI level. Table S2: Class separability and classification performance of the four RGB vegetation indices at the pixel level, global Youden-optimal threshold. Table S3: Linear regression between vessel-level area estimates, single global vs. concentration-specific thresholds. Table S4: Vessel-level Intersection over Union (IoU) between shoot-cluster masks obtained from the RGB-based and point-cloud methods, comparing the single global and concentration-specific threshold strategies. Table S5: Per-vessel area estimates for a separate external validation dataset, comparing RGB-based (ExG, GLI) and point-cloud-derived shoot area. Table S6: Classification performance of ExG and GLI at the fixed global Youden-optimal threshold evaluated separately within each sucrose treatment, pixel level. Table S7: Per-vessel performance of the fixed global thresholds. Table S8: Vessel-level mean index values underlying the Kruskal–Wallis test. Table S9: Leave-one-vessel-out cross-validation. Table S10: Validation of the global thresholds on the six vessels of the separate external dataset. Table S11: Shoot area of the six vessels of the separate external dataset, estimated from the index masks (GLI, ExG) and from the manually annotated masks. External dataset.zip Containing the shoot cluster polygons obtained from RGB vegetation indices (TF_index.zip) and from manual vectorization (TF_vector.zip). CSV.zip Containing the dataset used for the statistical analysis.

## Abbreviations

The following abbreviations are used in this manuscript:

AUC-ROC	Area Under the Receiver Operating Characteristic Curve
BG	Background
CI_red−edge_	Red-Edge Chlorophyll Index
DN	Digital Number
DSM	Digital Surface Model
ExG	Excess Green Index
FN	False Negative
FP	False Positive
GLI	Green Leaf Index
IoU	Intersection over Union
LiDAR	Light Detection and Ranging
MGRVI	Modified Green Red Vegetation Index
MTCI	MERIS Terrestrial Chlorophyll Index
NDVI	Normalized Difference Vegetation Index
RMSE	Root Mean Square Error
RGB	Red, Green, Blue
ROI	Region of Interest
ROC	Receiver Operating Characteristic
RTK	Real-Time Kinematic
SC	Shoot Cluster
SfM	Structure-from-Motion
SR	Separability Ratio
TN	True Negative
TP	True Positive
UAV	Unmanned Aerial Vehicle
VARI	Visible Atmospherically Resistant Index
