# Supplementary material for: Empirical Calibration of RGB Vegetation Indices for Non-Destructive Shoot Segmentation and Area Estimation in Blackberry Micropropagation
Source: Plants (Basel). 2026 Nov 6;15(21):3460. doi: 10.3390/plants15213460 (PMC13765689; doi:10.3390/plants15213460)
Supplement: Supplementary file 1 [file plants-15-03460-s001.zip › Supplementary Material S1.pdf]

Supplementary Materials: Empirical calibration of RGB vegetation indices for non-destructive shoot segmentation and area estimation in blackberry micropropagation

Raffaella Brigante[1], Laura Marconi[1], and Luca Regni[2*]

1. Supplementary Material

Descriptive statistics

Table S1. Descriptive statistics (mean ± SD) of the four RGB vegetation indices for the Shoot cluster and Background classes at the ROI level.

Class	ExG	GLI	MGRVI	VARI
SC (n = 182 ROIs)	83.04 ± 13.92	0.300 ± 0.048	0.227 ± 0.071	0.139 ± 0.046
BG (n = 12 ROIs)	12.07 ± 2.75	0.020 ± 0.005	0.002 ± 0.010	0.003 ± 0.010

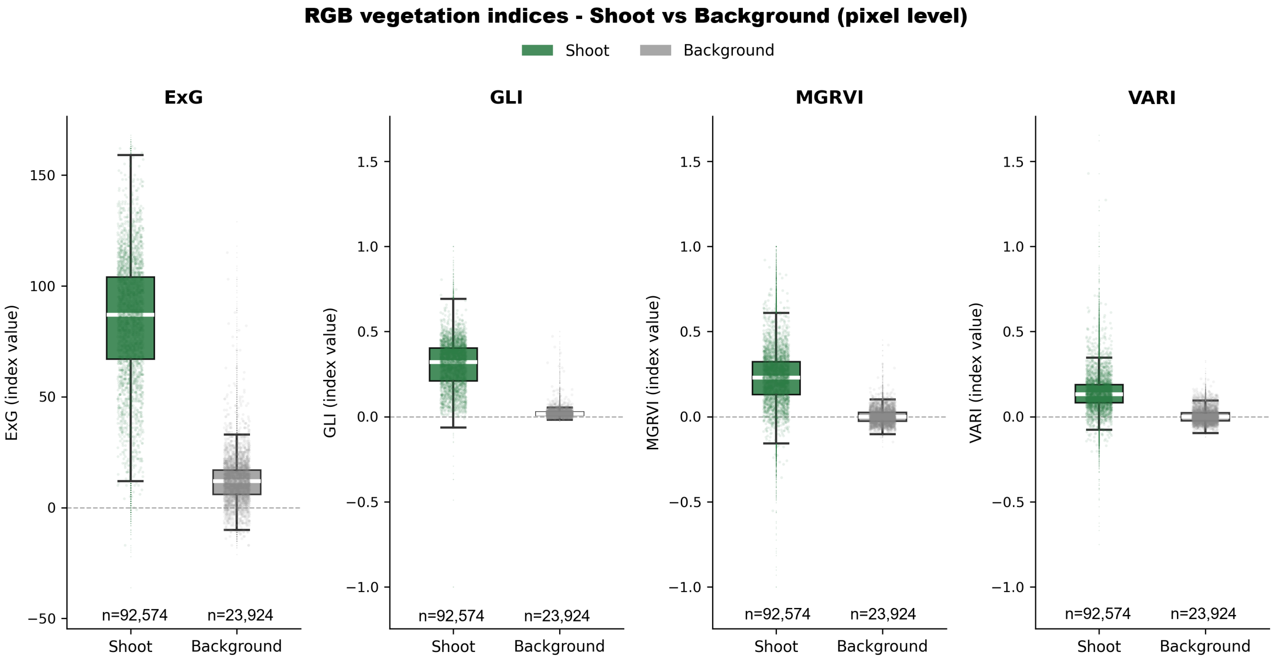

Figure S1. Box-plots of pixel-level values for the four RGB vegetation indices (ExG, GLI, MGRVI, VARI) by class (Shoot cluster: green, n = 92,574; Background: grey, n = 23,924). Boxes show the interquartile range (IQR); horizontal white lines indicate medians; whiskers extend to 1.5 × IQR. Jittered points represent a random subsample of 3,000 pixels per class. The y-axis is clipped to the 1st–99th percentile range for visual clarity only; all statistical analyses were performed on the full, unclipped pixel dataset, including outliers.

Classification performance metrics

Table S2. Class separability and classification performance of the four RGB vegetation indices at the pixel level, global Youden-optimal threshold.

Index	SR	AUC-ROC	Threshold	Sensitivity	Specificity	Accuracy	Precision	F1
GLI	1.879	0.9913	0.0658	0.9569	0.9812	0.9619	0.9949	0.9756
ExG	1.903	0.9879	33.0	0.9515	0.9727	0.9558	0.9927	0.9716
MGRVI	1.181	0.9381	0.0805	0.8422	0.9518	0.8647	0.9854	0.9082
VARI	0.981	0.9233	0.0524	0.8488	0.8881	0.8569	0.9671	0.9041

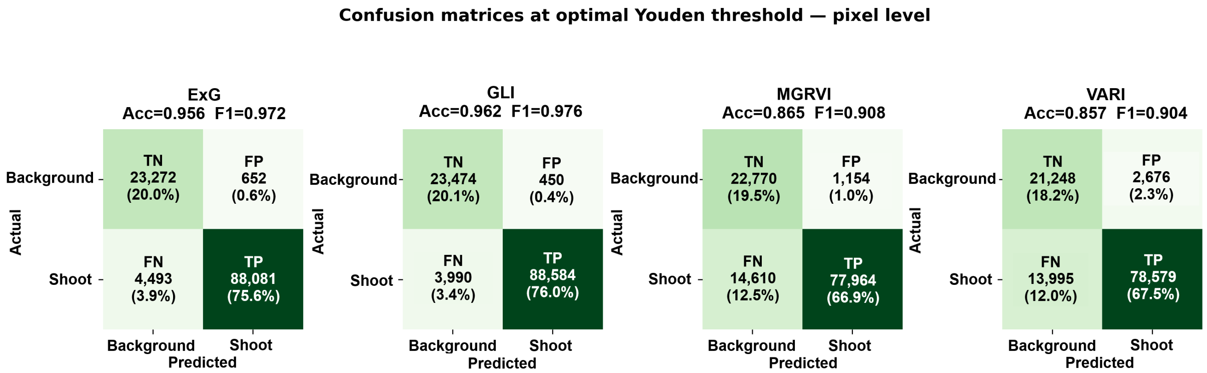

Figure S2. Confusion matrices for the four RGB vegetation indices at the optimal Youden threshold, pixel level. Cell values indicate absolute pixel counts and percentages of total pixels. TN: true negatives; FP: false positives; FN: false negatives; TP: true positives.

Concentration-specific threshold validation

The following tables and figures provide additional detail on the comparison between the single global threshold and concentration-specific thresholds, supporting the main conclusion that the single global threshold is sufficient for routine use.

Table S3. Linear regression between vessel-level area estimates ($n = 12$), single global vs. concentration-specific thresholds.

Comparison	Threshold	R^2	RMSE (cm^2)	Bias (cm^2)	RMSE$_{BA}$ (cm^2)	MAE (cm^2)
GLI vs Point Cloud	Single	0.990	0.64	+0.20	0.76	0.59
GLI vs Point Cloud	Concentration-specific	0.998	0.28	+0.44	0.57	0.50
ExG vs Point Cloud	Single	0.967	1.16	+0.43	1.40	1.12
ExG vs Point Cloud	Concentration-specific	0.996	0.39	+0.24	0.55	0.51
GLI vs ExG	Single	0.993	0.58	+0.24	–	–

RMSE is the root mean squared residual (divisor $n = 12$) of the OLS regression of point-cloud area on RGB-derived area (for the GLI vs ExG row, ExG area on GLI area). Bias is the mean paired difference RGB − Point Cloud (for the GLI vs ExG row, ExG − GLI), consistent with the Bland–Altman analysis. RMSE$_{BA}$ and MAE are computed directly on the same paired differences d (RMSE$_{BA}$ $= \sqrt{\text{mean}(d^2)}$, MAE $= \text{mean}(|d|)$); both are $\geq |\text{Bias}|$ in all rows, and RMSE$_{BA}$ is also $\geq$ the regression RMSE, whereas MAE can be smaller. Concentration-specific regression was not computed for the GLI vs ExG comparison.

Bland-Altman analysis (Fig. S3) confirmed good agreement between RGB-based and point cloud area estimates. GLI showed a small systematic bias of +0.20 cm^2 relative to the point cloud, with 95% limits of agreement (LoA) of $[-1.29, 1.69]$ cm^2 and 91.7% of vessels falling within these limits; ExG showed a slightly larger bias of +0.43 cm^2 (LoA: $[-2.29, 3.16]$ cm^2, 91.7% of vessels within limits). No significant proportional bias was detected for either comparison (GLI: $p = 0.167$; ExG: $p = 0.178$), indicating that measurement agreement did not depend on shoot area magnitude.

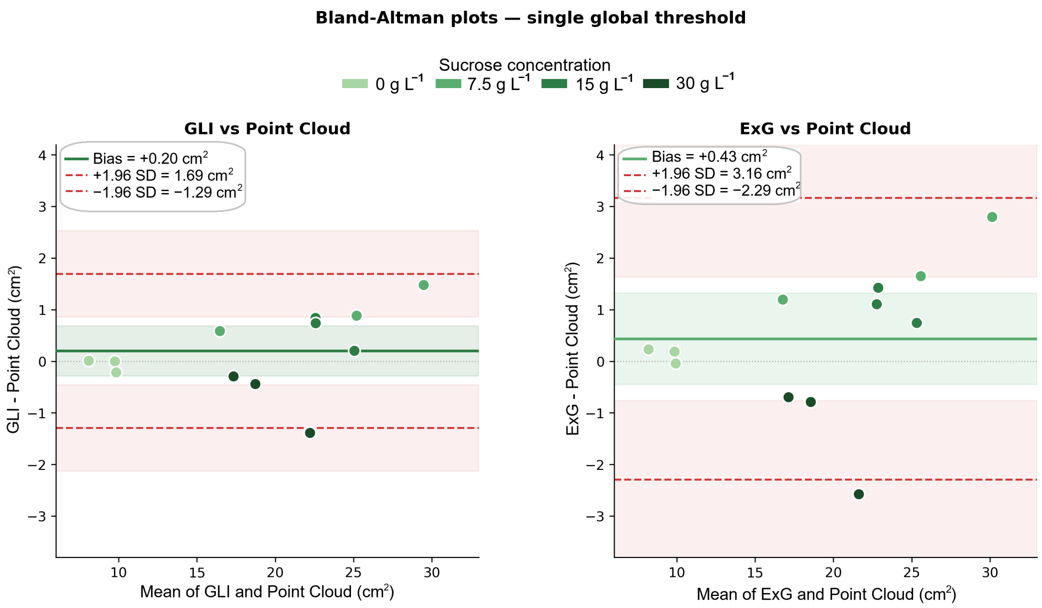

Figure S3. Bland–Altman plots comparing vessel-level shoot area estimates obtained from RGB-based segmentation (GLI and ExG) with the corresponding point-cloud reference ($n = 12$ vessels). Solid line: mean bias (RGB − Point Cloud); dashed lines: 95% limits of agreement (bias ± 1.96 SD). Points are coloured by sucrose concentration.

Table S4. Vessel-level Intersection over Union (IoU) between shoot-cluster masks obtained from the RGB-based and point-cloud methods, comparing the single global and concentration-specific threshold strategies.

Comparison	Threshold	Mean IoU	SD	Min	% > 0.8
ExG vs GLI	Single	0.957	0.014	0.933	100.0
GLI vs Point Cloud	Single	0.908	0.013	0.889	100.0
GLI vs Point Cloud	Concentration-specific	0.905	0.022	0.860	100.0
ExG vs Point Cloud	Single	0.888	0.024	0.848	100.0
ExG vs Point Cloud	Concentration-specific	0.898	0.027	0.860	100.0

Concentration-specific IoU was not computed for the ExG vs GLI comparison.

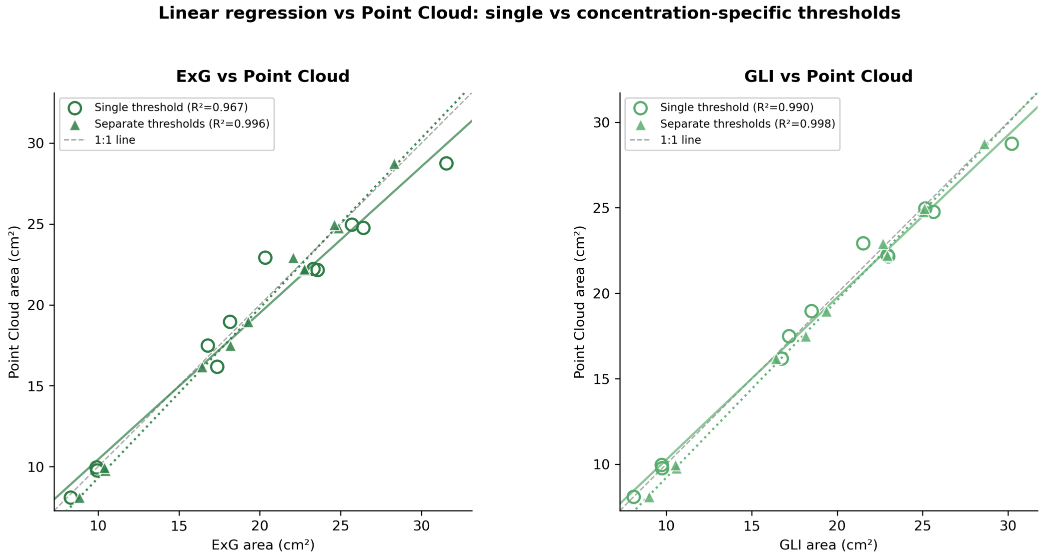

Figure S4. Linear regression between RGB-based and point cloud area estimates, comparing the single global threshold (open circles, solid regression line) and concentration-specific thresholds (filled triangles, dotted regression line) for ExG (left) and GLI (right). Grey dashed line: 1:1 identity line.

Table S5. Per-vessel area estimates for a separate external validation dataset (n = 6 vessels), comparing RGB-based (ExG, GLI) and point cloud-derived shoot area.

Vessel	Explants	Point Cloud (cm^2)	ExG (cm^2)	GLI (cm^2)	ΔExG (cm^2)	ΔGLI (cm^2)
TF-15A	15	8.719	8.205	8.411	−0.514	−0.308
TF-15B	15	7.075	7.604	7.388	+0.529	+0.312
TF-15C	15	10.298	10.070	9.978	−0.228	−0.320
TF-10A	10	6.770	6.390	6.445	−0.379	−0.324
TF-10B	10	6.698	5.904	6.015	−0.794	−0.683
TF-10C	10	6.821	6.045	6.053	−0.775	−0.768

Δ = RGB-derived area minus point cloud area (RGB − PC).

Table S6. Classification performance of ExG and GLI at the fixed global Youden-optimal threshold (ExG: $\theta = 33.0$; GLI: $\theta = 0.0658$), evaluated separately within each sucrose treatment, pixel level. IoU_{pixel} is computed from the sampled pixels; the whole-vessel mask overlap (IoU_{mask}) is reported in Table S7.

Sucrose (g/L)	Index	N points	Accuracy	Sensitivity	Specificity	IoU_{pixel}
0	ExG	30,000	0.968	0.962	0.993	0.960
0	GLI	30,000	0.964	0.956	0.995	0.955
7.5	ExG	28,500	0.975	0.983	0.947	0.969
7.5	GLI	28,500	0.975	0.978	0.967	0.969
15	ExG	28,500	0.966	0.966	0.965	0.957
15	GLI	28,500	0.965	0.962	0.976	0.956
30	ExG	29,498	0.915	0.897	0.986	0.893
30	GLI	29,498	0.944	0.933	0.987	0.929

Table S7. Per-vessel performance of the fixed global thresholds (GLI: $\theta = 0.0658$; ExG: $\theta = 33.0$). Sensitivity, specificity and point-based IoU (IoU_{pixel}) are computed on the sampling points of each vessel (pixel level); geometric IoU (IoU_{mask}) is computed over the complete vessel masks against the point-cloud-derived mask.

	GLI				ExG			
Vessel	Sens.	Spec.	IoU_{pixel}	IoU_{mask}	Sens.	Spec.	IoU_{pixel}	IoU_{mask}
0A	0.987	0.994	0.985	0.923	0.989	0.991	0.987	0.921
0B	0.935	0.996	0.934	0.901	0.945	0.995	0.943	0.901
0C	0.940	0.994	0.939	0.891	0.947	0.993	0.945	0.886
7.5A	0.970	0.942	0.956	0.904	0.976	0.914	0.954	0.876
7.5B	0.982	0.974	0.975	0.928	0.986	0.958	0.975	0.911
7.5C	0.981	0.984	0.977	0.927	0.987	0.970	0.979	0.907
15A	0.957	0.978	0.951	0.919	0.956	0.962	0.947	0.901
15B	0.965	0.977	0.960	0.909	0.971	0.967	0.963	0.896
15C	0.965	0.974	0.958	0.905	0.971	0.966	0.962	0.898
30A	0.946	0.984	0.943	0.900	0.916	0.982	0.912	0.850
30B	0.913	0.990	0.911	0.899	0.865	0.990	0.862	0.848
30C	0.936	0.986	0.933	0.889	0.907	0.984	0.903	0.862

Table S8. Vessel-level mean index values (mean of 15 Shoot ROIs per vessel, 17 in TF-30A) underlying the Kruskal-Wallis test of Section.

Vessel	Sucrose (g/L)	ExG	GLI	MGRVI	VARI
TF-0A	0	92.825	0.3039	0.2217	0.1308
TF-0B	0	89.974	0.2899	0.1777	0.1021
TF-0C	0	90.002	0.2779	0.1753	0.1018
TF-7.5A	7.5	88.033	0.3292	0.2757	0.1668
TF-7.5B	7.5	91.754	0.3580	0.3077	0.1823
TF-7.5C	7.5	96.537	0.3361	0.2758	0.1667
TF-15A	15	78.279	0.2950	0.2252	0.1384
TF-15B	15	83.050	0.2962	0.2282	0.1423
TF-15C	15	84.610	0.3107	0.2545	0.1600
TF-30A	30	72.025	0.2768	0.1912	0.1198
TF-30B	30	64.329	0.2654	0.1904	0.1206
TF-30C	30	66.475	0.2596	0.2080	0.1386

Table S9. Leave-one-vessel-out cross-validation: per-fold Youden-optimal threshold and held-out classification performance (pixel level).

Held-out vessel	Index	Threshold	Accuracy	Sensitivity	Specificity	IoU
TF-0A	ExG	33.000	0.989	0.989	0.991	0.987
TF-0A	GLI	0.0658	0.988	0.987	0.994	0.985
TF-0B	ExG	33.000	0.955	0.945	0.995	0.943
TF-0B	GLI	0.0658	0.948	0.935	0.996	0.934
TF-0C	ExG	33.000	0.956	0.947	0.993	0.945
TF-0C	GLI	0.0658	0.952	0.940	0.994	0.939
TF-7.5A	ExG	32.000	0.961	0.978	0.899	0.952
TF-7.5A	GLI	0.0609	0.964	0.976	0.919	0.955
TF-7.5B	ExG	33.000	0.980	0.986	0.959	0.975
TF-7.5B	GLI	0.0605	0.979	0.984	0.961	0.974
TF-7.5C	ExG	33.000	0.983	0.987	0.970	0.979
TF-7.5C	GLI	0.0657	0.981	0.981	0.983	0.977
TF-15A	ExG	33.000	0.957	0.956	0.963	0.947
TF-15A	GLI	0.0611	0.964	0.964	0.964	0.954
TF-15B	ExG	33.000	0.971	0.971	0.967	0.963
TF-15B	GLI	0.0658	0.968	0.965	0.977	0.960
TF-15C	ExG	33.000	0.970	0.971	0.966	0.962
TF-15C	GLI	0.0658	0.967	0.965	0.974	0.958
TF-30A	ExG	33.000	0.928	0.916	0.983	0.912
TF-30A	GLI	0.0658	0.954	0.946	0.985	0.943
TF-30B	ExG	33.000	0.891	0.865	0.990	0.862
TF-30B	GLI	0.0658	0.929	0.913	0.991	0.911
TF-30C	ExG	33.000	0.923	0.907	0.985	0.903
TF-30C	GLI	0.0658	0.947	0.936	0.986	0.933

Table S10. Validation of the global thresholds (GLI θ = 0.0658; ExG θ = 33.0) on the six vessels of the separate external dataset (blackberry "Thornfree"]) against manually annotated shoot masks. IoU, sensitivity and specificity were computed on the whole mask (area-based).

**Vessel**	**IoU**		**Sensitivity**		**Specificity**	
	**GLI**	**ExG**	**GLI**	**ExG**	**GLI**	**ExG**
TF-10A	0.921	0.888	0.962	0.940	0.991	0.988
TF-10B	0.908	0.872	0.943	0.915	0.993	0.991
TF-10C	0.897	0.856	0.933	0.910	0.993	0.989
TF-15A	0.906	0.844	0.941	0.896	0.994	0.991
TF-15B	0.906	0.870	0.963	0.957	0.991	0.986
TF-15C	0.910	0.838	0.948	0.912	0.992	0.983
Mean	0.908	0.861	0.949	0.921	0.992	0.988
Min	0.897	0.838	0.933	0.896	0.991	0.983
Max	0.921	0.888	0.963	0.957	0.994	0.991

IoU = TP/(TP+FP+FN); sensitivity = TP/(TP+FN); specificity = TN/(TN+FP). Specificity is computed within the bounding box enclosing the union of the manual, GLI and ExG masks of each vessel.

Table S11. Shoot area (cm^2) of the six vessels of the separate external dataset (blackberry "Thornfree"]), estimated from the index masks (GLI, ExG) and from the manually annotated masks, with bias (RGB − manual) in absolute (cm^2) and relative (%) terms.

**Vessel**	**Manual**	**Index area**		**Bias (cm^2)**		**Bias (%)**	
	**area**	**GLI**	**ExG**	**GLI**	**ExG**	**GLI**	**ExG**
TF-10A	6.41	6.45	6.39	+0.04	−0.02	+0.62	−0.30
TF-10B	6.13	6.01	5.90	−0.11	−0.22	−1.80	−3.61
TF-10C	6.22	6.05	6.05	−0.16	−0.17	−2.62	−2.76
TF-15A	8.58	8.41	8.21	−0.17	−0.38	−1.97	−4.37
TF-15B	7.20	7.39	7.60	+0.19	+0.41	+2.66	+5.67
TF-15C	10.08	9.98	10.07	−0.10	−0.01	−0.97	−0.06
Mean	7.43	7.38	7.37	−0.05	−0.06	−0.68	−0.91
Min	6.13	6.01	5.90	−0.17	−0.38	−2.62	−4.37
Max	10.08	9.98	10.07	+0.19	+0.41	+2.66	+5.67
Mean $\lvert$bias$\rvert$				0.13	0.20	1.77	2.79

Bias = area from RGB index mask − area from manual mask; relative bias = bias/manual area × 100. Min and Max refer to signed values.

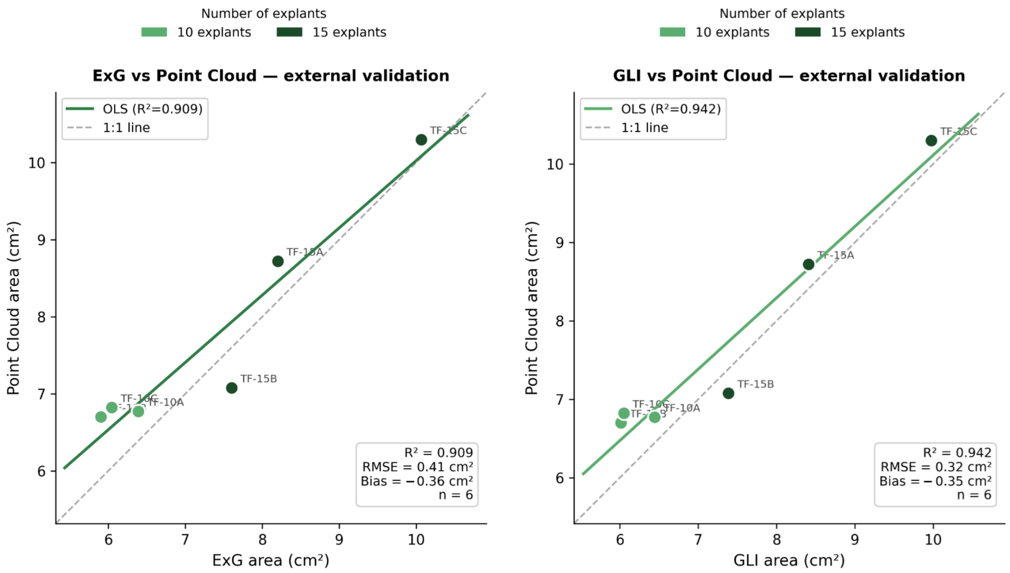

Figure S5. Linear regression between RGB-based (ExG, left; GLI, right) and point-cloud-derived shoot area estimates for the external dataset ($n = 6$ vessels). Points are coloured by number of explants (light green: 10; dark green: 15). Green solid line: OLS fit; grey dashed line: 1:1 identity line. Global thresholds calibrated on the main dataset (ExG: $\theta = 33.0$; GLI: $\theta = 0.0658$) were applied without recalibration.

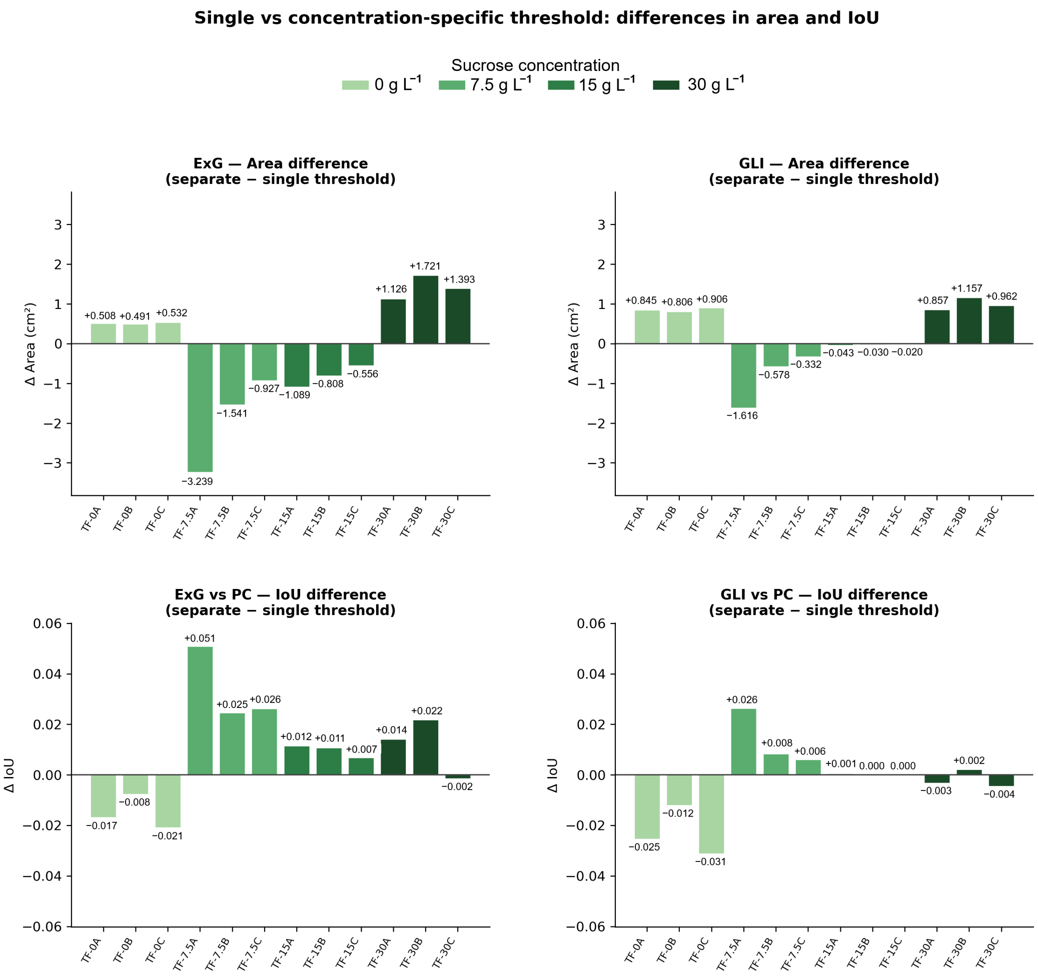

Figure S6. Differences between concentration-specific and single global threshold approaches. Top row: area differences (cm^2) for ExG (left) and GLI (right) by vessel. Bottom row: IoU differences relative to Point Cloud for ExG (left) and GLI (right) by vessel. Bars are coloured by sucrose concentration; positive values indicate larger area/IoU under the concentration-specific threshold.

https://doi.org/10.3390/plants1010000
